# Cascades of Genetic Instability Resulting from Compromised Break-Induced Replication

**DOI:** 10.1371/journal.pgen.1004119

**Published:** 2014-02-27

**Authors:** Soumini Vasan, Angela Deem, Sreejith Ramakrishnan, Juan Lucas Argueso, Anna Malkova

**Affiliations:** 1 Department of Biology, School of Science, IUPUI, Indianapolis, Indiana, United States of America; 2 Department of Environmental and Radiological Health Sciences Colorado State University, Fort Collins, Colorado, United States of America; 3 Department of Biology, College of Liberal Arts and Sciences, University of Iowa, Iowa City, Iowa, United States of America; Baylor College of Medicine, United States of America

## Abstract

Break-induced replication (BIR) is a mechanism to repair double-strand breaks (DSBs) that possess only a single end that can find homology in the genome. This situation can result from the collapse of replication forks or telomere erosion. BIR frequently produces various genetic instabilities including mutations, loss of heterozygosity, deletions, duplications, and template switching that can result in copy-number variations (CNVs). An important type of genomic rearrangement specifically linked to BIR is half-crossovers (HCs), which result from fusions between parts of recombining chromosomes. Because HC formation produces a fused molecule as well as a broken chromosome fragment, these events could be highly destabilizing. Here we demonstrate that HC formation results from the interruption of BIR caused by a damaged template, defective replisome or premature onset of mitosis. Additionally, we document that checkpoint failure promotes channeling of BIR into half-crossover-initiated instability cascades (HCC) that resemble cycles of non-reciprocal translocations (NRTs) previously described in human tumors. We postulate that HCs represent a potent source of genetic destabilization with significant consequences that mimic those observed in human diseases, including cancer.

## Author Summary

Maintaining genomic stability is important to prevent birth defects, genetic disorders and other diseases, including cancer. Double-strand DNA breaks (DSBs), which can result from exposure of living cells to ionizing radiation and various chemicals, threaten genomic integrity, thus making DSB repair essential. The choice of DSB repair pathway is important because some pathways confer destabilizing consequences. Break-induced replication (BIR) is a mechanism of DSB repair that is often associated with deleterious events that can threaten genetic stability. One such deleterious event is the formation of half-crossovers (HCs), which occurs when two chromosomes physically interacting during BIR repair fuse. Here we employed a yeast-based system to unravel the genetic factors promoting HC formation. We demonstrate that the interruption of BIR due to problems in DNA synthesis or checkpoint control, promote HCs. Additionally, we document that disruption of BIR promotes half-crossover-initiated cascades (HCC) that can significantly destabilize the genome and could be accounted as a potential mechanism responsible for cycles of non-reciprocal translocations contributing to cancer in humans.

## Introduction

Double-strand DNA breaks (DSBs) are dangerous because they can lead to chromosomal rearrangements or cell death. DSBs may result from a cell's exposure to various DNA-damaging agents, such as radiation and various chemicals, including anti-cancer drugs. In addition, problems with DNA metabolism can also result in DSB formation. DSB-induced changes to the genome have been implicated in promoting various human diseases, including cancer, which emphasizes the importance of proper repair of such lesions. Multiple pathways of DSB repair have evolved (reviewed in [1], [2]). Non-homologous end joining (NHEJ) is a repair mechanism in which two non-homologous broken ends of a DNA molecule fuse together, often producing small DNA insertions and deletions that can be destabilizing. Alternatively, homologous recombination (HR) mechanisms repair DSBs through recombination, where broken DNA ends initiate copying of a homologous sequence elsewhere within the genome. The most efficient pathway of HR is gene conversion (GC), where both ends of a DSB use a homologous sequence to copy lost DNA in order to repair the DSB lesion. Alternatively, break-induced replication (BIR) is an HR mechanism that employs only a single end of a DSB for repair.

During BIR, a single broken DNA end invades a homologous region within the genome to initiate extensive DNA synthesis that can copy large portions of a donor molecule through its telomere (reviewed in [3]–[6]). BIR is a primary pathway to repair broken replication forks and eroded telomeres. Also, it has been observed that gap repair can proceed through BIR [7] and, for reasons that are not entirely clear yet; the frequency of BIR is known to increase in aged cells [8]. BIR is initiated by strand invasion, which occurs with kinetics similar to those of the GC pathway [7]. However, after strand invasion, progress stalls and DNA synthesis is delayed by 4 or more hours [9]. The exact reason for this pause is not known, but several possibilities have been proposed, including slow replication fork assembly, unstable D-loop formation, and the existence of a “recombination checkpoint”, discouraging BIR repair (reviewed in [3], [5], [6]). The delay in BIR initiation leads to the establishment of a checkpoint-mediated G2/M arrest, which allows cells to complete BIR prior to cell division. Consistently, it was observed that a defective checkpoint (achieved by deletion of *RAD9*) decreased BIR efficiency and also increased chromosome loss [9]. Once DNA synthesis associated with BIR is initiated, it is fast and processive, similar to normal S-phase DNA replication [9]. It has been demonstrated that the initiation of BIR DNA synthesis involves the majority of proteins required for initiation of S-phase DNA replication [10]. Also, Polδ, a main replicative polymerase, plays a crucial role in DNA synthesis in BIR [11]–[13]. However, the role of two other replicative polymerases, Polε and Polα, in BIR remains unclear [11], (also reviewed in [5], [6]). In addition, the mechanism of BIR is drastically different from S-phase replication as it proceeds via migrating DNA bubble leading to conservative inheritance of newly synthesized strands [14], [15], [16].

While the end result of BIR is repair of the DSBs, the mechanism of BIR increases the likelihood of a variety of deleterious outcomes that may have destabilizing consequences in the genome. Among these are loss of heterozygosity, deletions, duplications, translocations, copy-number variations, and a significantly elevated mutation rate [9], [17]–[25]. In addition, half- crossovers (HCs), which are chromosome fusions initially identified in *rad51*Δ and *rad52*Δ mutants [13], [26]–[29], were recently demonstrated to occur in wild type and various mutants following initiation of BIR [12], [13]. BIR-induced HC formation is initiated by strand invasion, but the resulting intermediate ruptures prior to repair to yield a rearranged chromosome consisting of fused pieces of the recipient and donor molecules, as well as a destabilized fragment of the broken donor. Accordingly, HC formation requires proteins involved in the strand invasion step of BIR, however the impairment of proteins involved in BIR after strand invasion promotes HCs [12]. Thus, HCs are markedly elevated in yeast bearing mutations *pol32Δ* and *pol3-ct*, which interfere with successful initiation of DNA synthesis [12], [13]. It was proposed that the failure to initiate DNA synthesis in these mutants promotes resolution of the Holliday junction (HJ) formed during strand invasion. The exact mechanism of HJ resolution remains unknown, though the resolvase Mus81 has been implicated as one protein capable of resolving HJs and therefore may contribute to HC formation [13].

HCs result in the breakage of a previously intact donor chromosome, and this can have deleterious consequences by initiating recurrent cycles of genetic instability. Analogous cycles (called NRTs, for non-reciprocal translocations) have been described in mammalian tumors where broken chromosomes initiate recombination with an intact donor, which in turn leads to breakage of the donor [30]. While the molecular mechanism of NRTs remains undefined, we have previously proposed [12] that cycles of NRTs are mediated by cascades of HCs that continue until the donor fragments are either stabilized or lost. Thus, further investigation into HC formation and the possible cascades of genetic instability that may result is warranted.

To further define mechanisms of HC formation and the effects of HCs on genetic instability, we hypothesized that various factors that interrupt ongoing BIR replication may induce HC formation in a manner similar to mutations that prevent initiation of BIR replication. We show that interruption of BIR synthesis by exposure of cells to DNA damaging agents or due to a defective replisome results in a dramatic increase in HCs. Moreover we demonstrate that a disruption of BIR imposed by premature onset of mitosis increase HC formation. Finally, we document the occurrence of half-crossover-initiated instability cascades (HCCs) that closely resemble NRT cycles observed in cancer cells.

## Results

### Experimental system

Half-crossovers (HCs) are chromosome fusions resulting from aberrant processing of BIR intermediates. It has been proposed that HCs lead to deleterious consequences by initiating cascades of genetic instabilities. Here we aimed to identify genetic factors that promote the channeling of BIR into HC formation.

**Figure 1:**
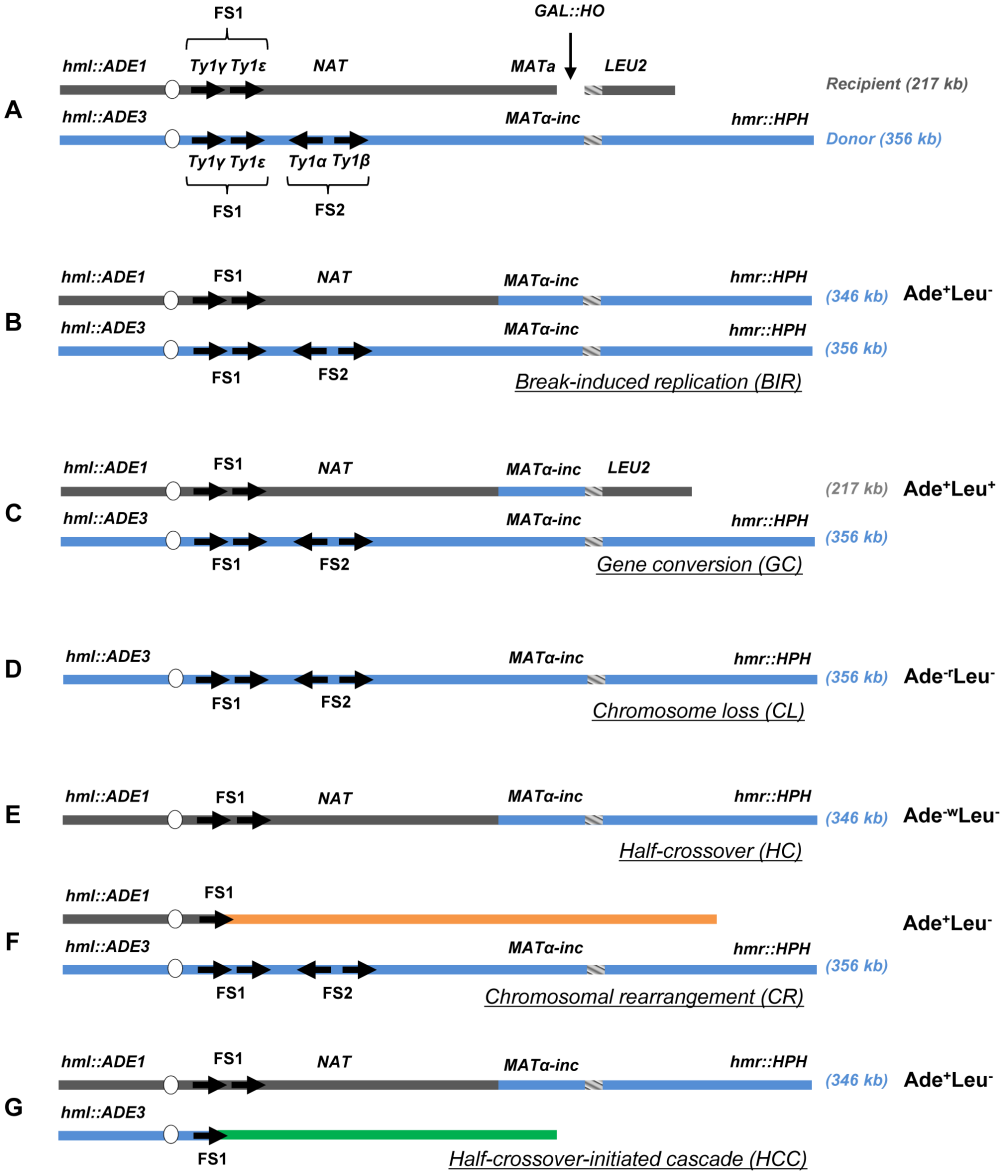
Experimental system to study BIR and half-crossover formation. (A) Strain disomic for Chromosome (Chr) III (AM1003) [12]. A DSB is created at *MAT*a in truncated Chr III (recipient (upper) chromosome) by a galactose-inducible *HO* endonuclease. The *MAT*α-inc chromosome (donor (lower) chromosome) is full-length and is resistant to cutting by *HO*. The ends of the recombining chromosomes are marked by *ADE1*, *LEU2*, *ADE3* and *HPH*. Two copies of Ty1 elements (Ty1α and Ty1β), comprising the FS2 region located ∼30 kb centromere proximal from *MAT*a, are replaced by a *NAT* cassette in the recipient chromosome. The positions of two other Ty elements (Ty1γ and Ty1ε) comprising the FS1 region are shown. (B) Schematic representation of an Ade^+^Leu^−^ (Break-Induced Replication (BIR)) outcome. (C) Ade^+^Leu^+^ (Gene Conversion (GC)) outcome. (D) Ade^−r^Leu^−^ (Chromosome Loss (CL)) outcome. (E) Ade^−w^Leu^−^ (Half-Crossover (HC)) outcome. (F) Ade^+^Leu^−^ (Chromosomal rearrangement (CR)) outcome. (G) Ade^+^Leu^−^ (Half-crossover-initiated cascade (HCC)) outcome.

To assay the efficiency of BIR and the frequency of half-crossovers in DSB repair, we employed our disomic experimental system in yeast, *Saccharomyces cerevisiae*, wherein a galactose-induced DSB is initiated at the *MAT*a locus on the truncated copy of chromosome III (recipient chromosome) (Fig. 1A) [12]. The second full copy of chromosome III contains the uncleavable *MATα*-inc allele and serves as a template for DSB repair (donor chromosome). Upon induction of the DSB, DNA is repaired predominantly by BIR (Fig. 1B) because only one end of the DSB has large homology to the full-length donor copy of chromosome III. The ends of both chromosomes that participate in BIR repair are marked by *ADE1*, *LEU2*, *ADE3* or *HPH*; such that repair outcomes can be determined using appropriate selective media. Also, a *NAT* cassette was used to replace a region 30 kb centromere-proximal to *MAT*a that contained two Ty1 elements (FS2) in the recipient chromosome [21]. Using these genetic markers, it was determined that more than 75% of DSB repair outcomes displayed an Ade^+^Leu^−^ phenotype, indicating BIR repair of the galactose-induced DSB (Fig. 1B, Fig. S1). Approximately 14% of the DSB repair outcomes were Ade^+^Leu^+^, indicating the DSB was repaired by gene conversion (GC) (Fig. 1C, Fig. S1). Other colonies had an Ade^−r^Leu^−^ phenotype (were *ADE1-*deficient and red (as described in [12]) and resulted from failure of the chromosome to repair the DSB leading to chromosome loss (CL) (Fig. 1D). Also, a small number of colonies were Ade^−w^Leu^−^ (were *ADE3*-deficient and white), which represented HC events resulting from fusion of the *ADE1*-containing segment of the recipient chromosome with the *HPH*-containing segment of the donor chromosome and concurrent loss of the *ADE3* and *LEU2* segments of the donor and recipient chromosomes, respectively (Fig. 1E).

### Reduced processivity of Polδ promotes HC formation

**Figure 2:**
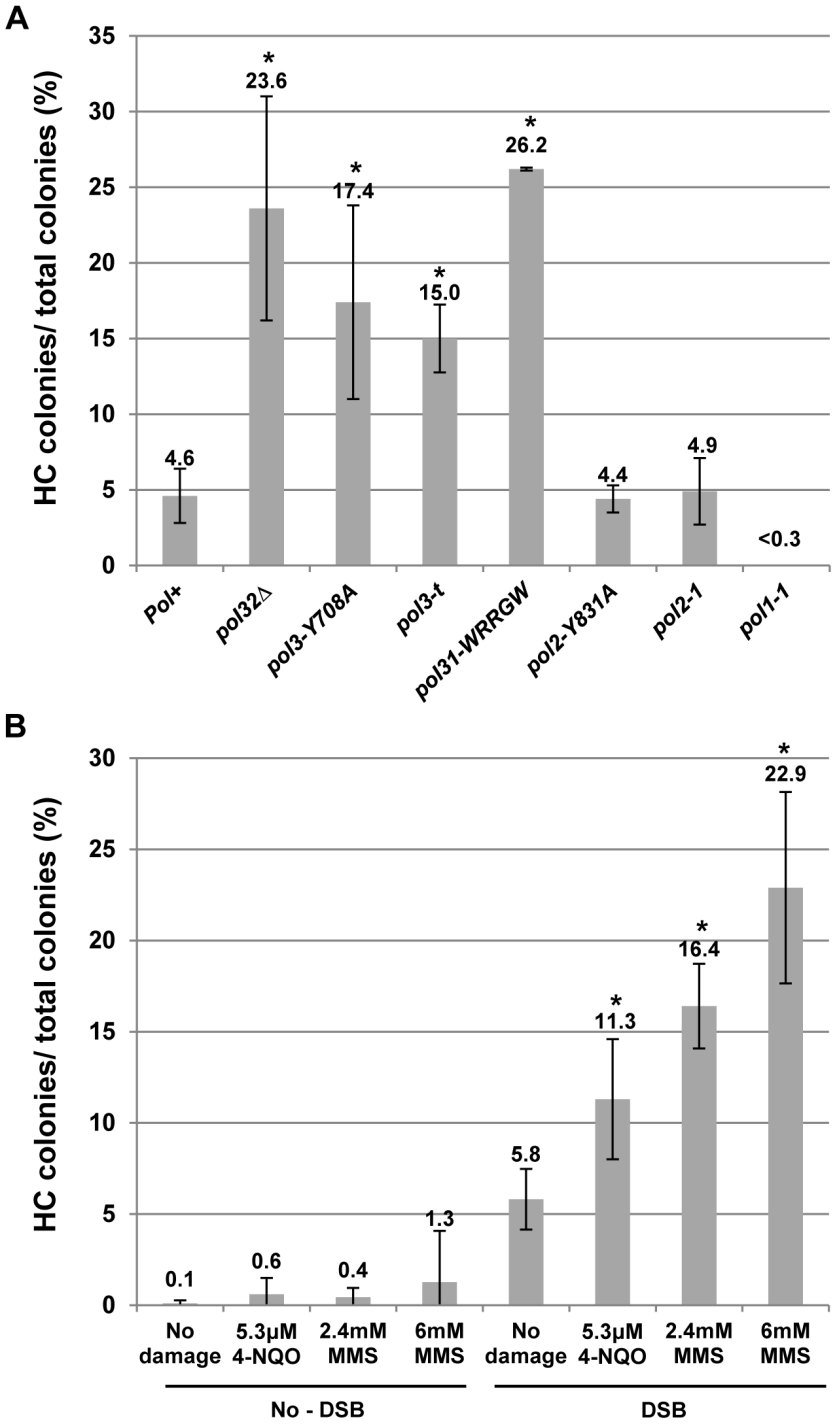
Effects of DNA damage and of replisome defect on half-crossover formation. (A) Effect of mutations impairing DNA polymerases on HC formation. Results of 3 to 14 experiments performed for each strain were used to calculate the average ± SD percent of colonies containing a HC. Asterisks indicate statistically significant increases compared to the Pol^+^ (wild type) strain. See the text for P-values. For the number of colonies analyzed, see Materials and Methods. The frequency of HCs in *pol32*Δ was presented previously [12]. Because no HC outcomes were observed in *pol1-1* strains among 454 analyzed colonies, we estimated that the frequency of colonies with HCs was less than 0.3%. (B) Effect of DNA damaging agents MMS (2.4 and 6 mM) and 4-NQO (5.3 µM) on HC formation. Results of 3 to 8 experiments performed for each strain were used to calculate the average ± SD percent of colonies containing a HC. Experiments performed in the absence of galactose represent no-DSB controls. Asterisks indicate statistically significant increases compared to the no-damage control.

We tested the effect of mutations that impair DNA polymerases on HC formation by plating yeast on a galactose-containing medium [12]. Although each of the Polδ mutations tested here had varying effects on BIR efficiency, they all stimulated HC formation. In particular, *pol3-Y708A*, a mutation that affects the catalytic subunit of Polδ [31], dramatically decreased BIR efficiency, and increased chromosome loss (P<0.0001; Fig. S1). Also, *pol3-Y708A* increased the number of colonies containing HCs to 17% compared to approximately 5% in wild type (P<0.0001; Fig. 2A; see also Fig. S1; note that Fig. 2 presents the fraction of colonies that are fully or partially HCs, while Fig. S1 shows the fraction of HCs among all DSB repair events). Similarly, the *pol31-WRRGW* mutation, which disrupts the Pol31-Pol32 interaction [32], displayed similar effects and elevated HCs to 26% (P<0.0001; Fig. 2A; Fig. S1). These phenotypes were similar to those previously observed in *pol32Δ* and *pol3-ct* mutants [12], [13], suggesting that HCs in these mutants are promoted predominantly by failure to initiate BIR. In strains bearing the *pol3-t* mutation known to compromise the processivity of Polδ during S-phase DNA replication [33], [34], HCs were also elevated (P<0.0001), even though these cells frequently successfully completed BIR repair (Fig. 2A; Fig. S1). Therefore, the increase of HCs in *pol3-t* might be explained by interruptions of ongoing BIR rather than by problems in BIR initiation. Thus, an intact Polδ appears to be necessary to prevent HC formation. Conversely, mutations impairing Polε showed no effect on HCs. Thus, no increase compared to wild type was observed in either *pol2-1* [35] mutants with a truncated catalytic subunit or in *pol2-Y831A* mutants [31] with a mutation in the same conserved catalytic motif as *pol3-Y708A* mutants (Fig. 2A). Interestingly, the *pol1-1* mutation [36], which impairs Polα (a part of the primase complex), decreased HCs to less than 0.3% (P<0.0001; Fig. 2A).

### DNA damage is synergistic to BIR in promoting HC formation

Due to the increased HCs observed in mutants with decreased polymerase processivity, we hypothesized that BIR pausing induced by damage in the template DNA used for BIR could promote HC formation. This idea was tested by analyzing DSB repair in our BIR system in the presence of DNA damaging agents. Following induction of BIR in galactose-containing liquid cultures, cells were exposed to either the alkylating agent MMS or 4-NQO (UV mimetic) for seven hours while BIR repair occurred. Cells were plated on YEPD and the resulting colonies were analyzed using selective media (Fig. 2B; Fig. S2). Cell viability was calculated by plating cells on YEPD (see Materials and Methods for details). Both drugs were deactivated (see Materials and Methods) prior to being plated on YEPD. In cells treated with 2.4 or 6 mM MMS, the percent of colonies with HC outcomes was increased approximately 3- and 4-fold, respectively, compared to cells treated with galactose but no drug (P<0.0001), while the percent of HC outcomes approximately doubled in cells treated with 5.3 µM 4-NQO compared to the same control (P<0.0001; Fig. 2B; see also Fig. S2). Significantly, the level of HCs in cells exposed to damage alone (without DSB induction) was not significantly increased in comparison to no-damage control (Fig. 2B, no-DSB). Thus, our data suggest that the observed increase in HCs is promoted not by DNA damage *per se*, but by base damage in the chromosomal region undergoing BIR repair. Our data also identifies a previously unknown synergy between BIR and DNA damage that dramatically increases the rate of HC formation. We note that the levels of DNA damaging drugs used in these experiments, as expected, decrease viability (see Materials and Methods). Overall, since both MMS and 4-NQO induce base damage capable of blocking DNA polymerases (and therefore replication) [37]–[39], our results suggest that interruption of ongoing BIR leads to the aberrant processing of BIR intermediates resulting in HC formation.

### Checkpoint deficiency promotes HC formation

**Figure 3:**
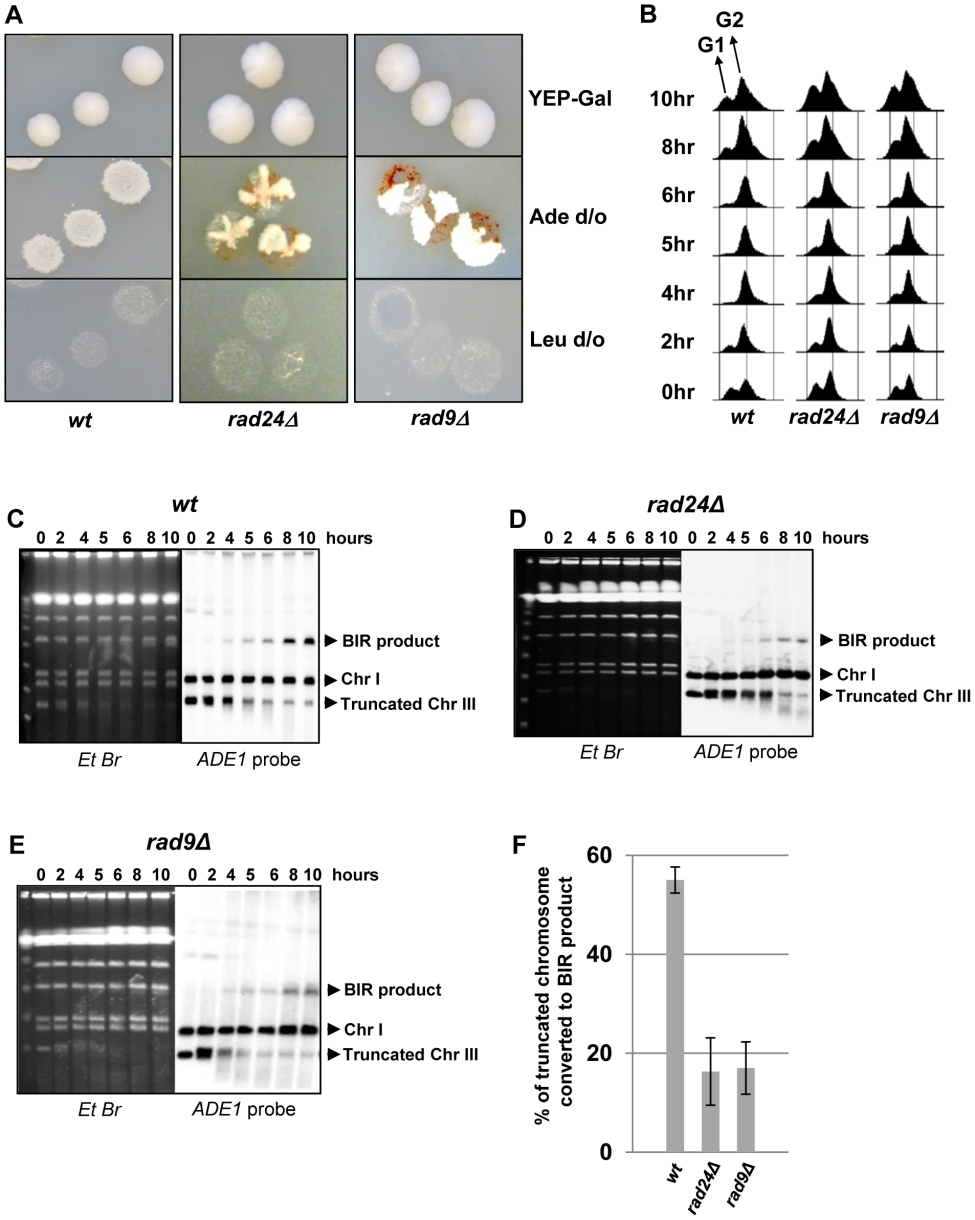
DSB repair in checkpoint-deficient mutants. (A) Colonies representing DSB repair outcomes in Rad^+^ (wild type; wt), *rad24*Δ, and *rad9*Δ cells. The morphology of colonies grown on YEP-Gal (top row) and following replica-plating on synthetic complete adenine drop-out medium (Ade d/o, middle row) and on leucine drop-out medium (Leu d/o, bottom row). (B) Cell cycle analysis by flow cytometry of cells undergoing BIR repair in wt, *rad24*Δ, and *rad9*Δ strains. Positions of peaks corresponding to G1 and G2 phases of the cell cycle are indicated. (C) BIR kinetics were analyzed by PFGE using cells removed at indicated time points following DSB induction (ethidium bromide- stained gel (left)) followed by Southern hybridization with an *ADE1*-specific probe (right) in wt (C), *rad24*Δ (D) and *rad9*Δ (E). (F) Quantification of BIR efficiency 10 hours after addition of galactose. Results of 3 experiments performed on each strain were used to calculate average ± SD efficiency of BIR (defined as percent of truncated chromosome converted to BIR product).

**Figure 4:**
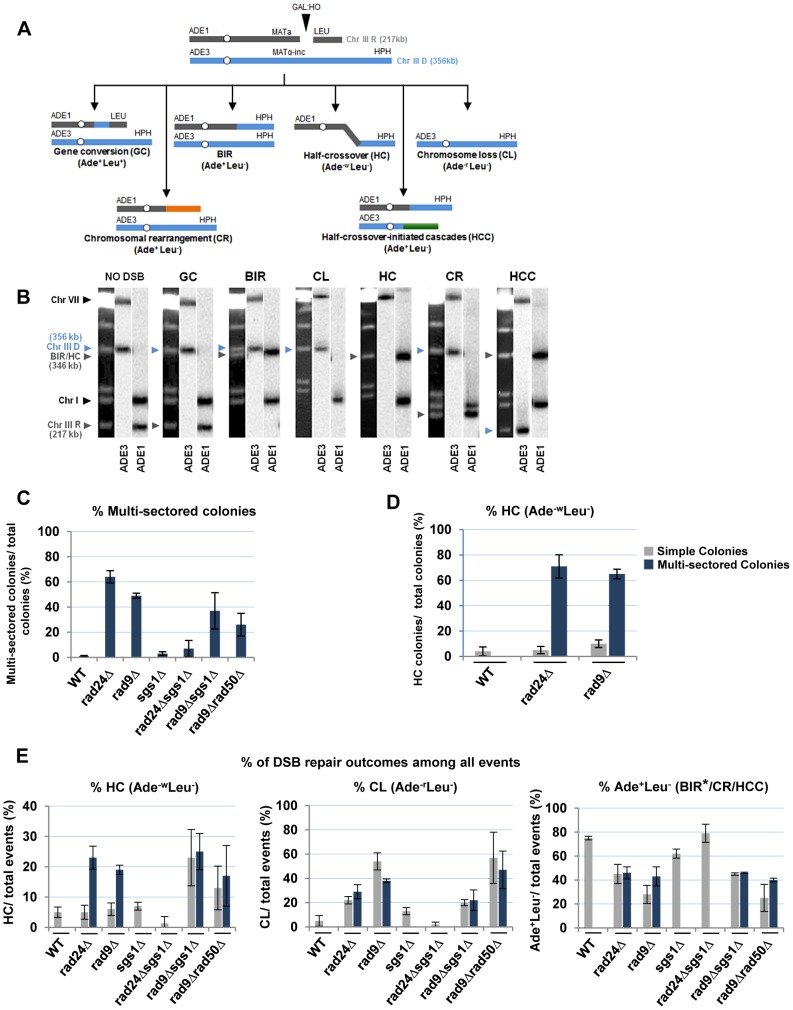
Distribution of repair outcomes in checkpoint-deficient mutants. (A) Schematic diagram depicting the following DSB repair outcomes: gene conversion (GC), break-induced replication (BIR), chromosome loss (CL), half-crossover (HC), chromosome rearrangement (CR) resulting from stabilization of the broken recipient chromosome via ectopic recombination or by *de novo* telomere formation, and half-crossover-initiated cascade (HCC) initiated by HC and resulting from stabilization of the broken donor chromosome. “R” and “D” indicate the recipient and the donor chromosomes, respectively. (B) PFGE analysis of the DSB repair outcomes listed in A. Light bands correspond to chromosomes stained with ethidium bromide, while dark bands correspond to hybridization with *ADE1* (recipient (R) -specific) or *ADE3* (donor (D)-specific) probes marked by grey and blue arrowheads, respectively. (C) Percentage of multi-sectored colonies (containing ≥3 repair sectors) formed following DSB induction in wt and checkpoint-deficient mutants. Results of 3 to 4 experiments performed for each strain were used to calculate the average ± SD. See Materials and Methods for the number of analyzed colonies. (D) Percent of colonies containing HC events in wt and checkpoint-deficient mutants. (E) Percent of various repair outcomes (HC, CL and Ade^+^Leu^−^) calculated among all repair events detected in simple and multi-sectored colonies (See Materials and Methods for details). Asterisk indicates that BIR could not be distinguished from cases where a HC chromosome segregated in mitosis with an intact donor chromosome (see text for details).

Previously, we demonstrated that initiation of DNA synthesis during BIR is a very slow process (takes up-to 4 hours) and leads to the establishment of a checkpoint-mediated G2/M cell cycle arrest [7], [9] that prevents mitotic division and thus allowing cells to complete BIR. Given our data that interruptions in BIR due to decreased processivity of polymerases or DNA damage promote HC formation, we hypothesized that checkpoint deficiency may stimulate HC formation due to an interruption in BIR progression by the premature onset of mitosis. To test this hypothesis, we analyzed BIR outcomes in mutants lacking Rad9 or Rad24, which are required for the DNA damage response (reviewed in [40]). Consistent with previous observations in these mutants [9], checkpoint deficiency led to a high frequency of multi-sectored colonies (colonies containing ≥3 different repair sectors) (Fig. 3A, Fig. 4C). Formation of multi-sectored colonies likely resulted from premature onset of mitosis, and DSB repair that occurred after subsequent cell divisions and took place only in a fraction of the daughter calls. Consistent with this idea were the results of FACS analyses that confirmed full G2/M arrest in wild type cells between 4 and 8 hours after DSB induction, with only partial arrest observed at these time points in *rad9Δ* and *rad24Δ* mutants (Fig. 3B).

We observed that the percentage of colonies with at least one HC sector was extremely high, 71% and 65% among multi-sectored colonies of *rad24Δ* and *rad9Δ* mutants respectively. This was a significant increase (P<0.0001) compared to wild type where the frequency of colonies with HC was only 4% (Fig. 4D). Also, when calculated as a fraction of all sectors, HC sectors comprised approximately 20% in *rad9Δ* and *rad24Δ* mutants (Fig. 4E; left). Among simple colonies (with no more than 2 sectors or events evident), no notable difference in HC frequency was observed between the checkpoint-deficient and wild type strains (Fig. 4D). We propose that the increase in HCs in checkpoint-deficient mutants results from premature onset of mitosis that may occur either during the first cell division following DSB induction or during subsequent cell divisions, as further explored in the following sections.

### DSB repair in checkpoint-deficient mutants results in colonies with multiple, complex outcomes

**Figure 5:**
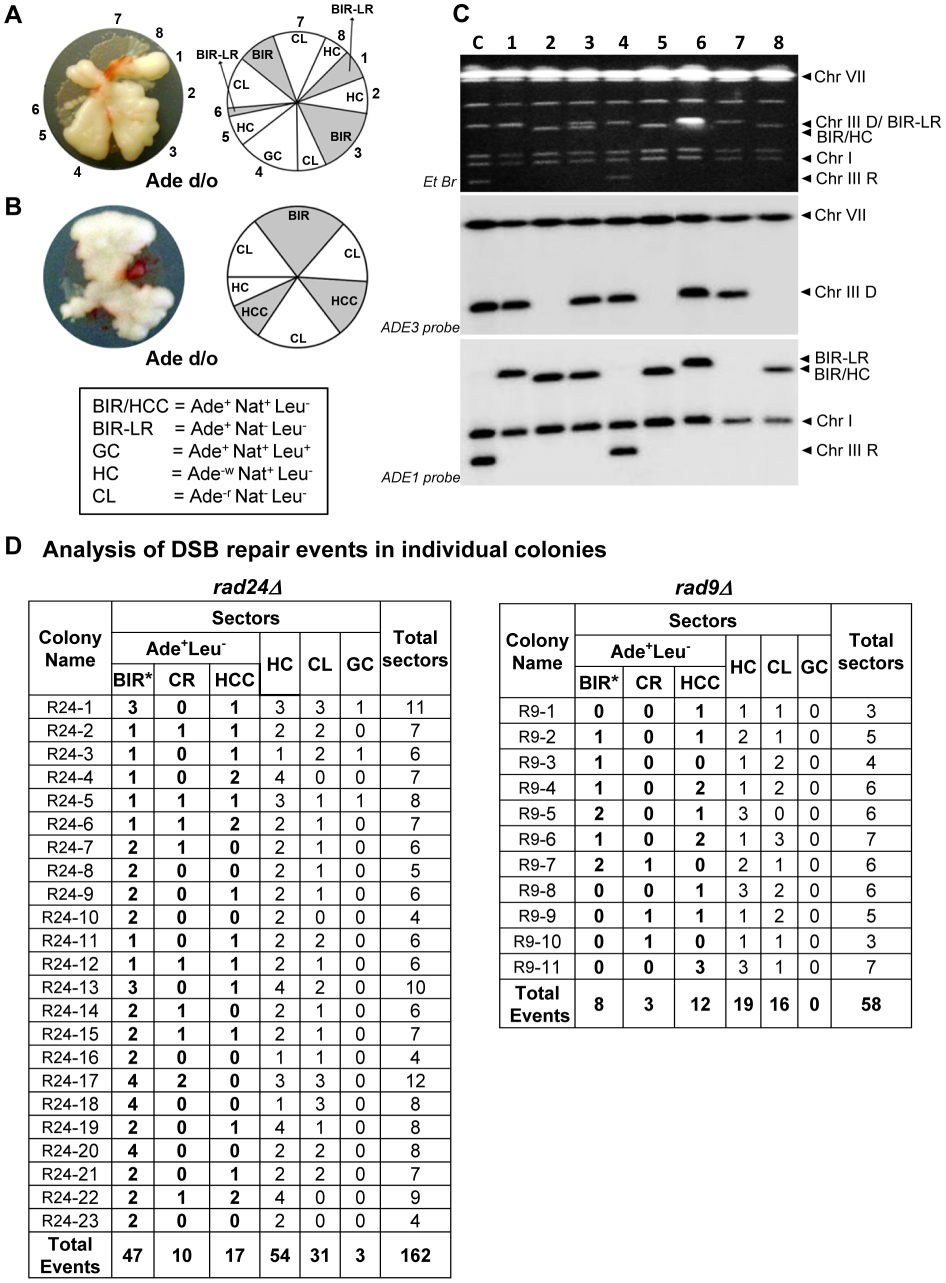
Analysis of individual colonies formed by checkpoint-deficient mutants following DSB induction. Analysis performed on colonies containing at least one HC sector. The representative colonies of (A) *rad24*Δ *and* (B) *rad9*Δare shown. Photograph (left) and schematic representation of all sectors (right) are presented. (C) PFGE analysis of all sectors from the colony shown in A. Lane labeled “C”: genomic DNA from *rad24*Δ before DSB induction. Lanes numbered 1–8 represent sectors from the colony in A. Top: Ethidium bromide-stained gel; middle and bottom: Southern blot hybridization with *ADE3*- and *ADE1*-specific probes, respectively. Note that the size of BIR products in BIR-LR outcomes (lanes 1 and 6) is 356 kb instead of 346 kb because they resulted from strand invasion centromere proximal to *NAT* (see Materials and Methods for details). (D) Summary of the results of PFGE analysis of all sectors from individual colonies in *rad24*Δ (left) and *rad9*Δ (right). All DSB repair outcomes are abbreviated similarly to Fig. 4A.

Genetic analysis of repair outcomes in checkpoint-deficient mutants revealed increased chromosome loss (P<0.0001; Fig. 4A, 4E; middle) and a decreased level of Ade^+^Leu^−^outcomes, which normally represent BIR (P<0.0001; Fig. 4A, 4E; right). This was consistent with our previous results [9] and most likely reflected failed DSB repair in these strains. The decreased BIR efficiency in checkpoint-deficient mutants was further supported by PFGE analysis of cells undergoing DSB repair over a 10-hour time course (performed similar to [12]), where the amount of BIR repair product was significantly reduced in *rad9*Δ and *rad24*Δ mutants compared to the wild type (Fig. 3C–F)). Based on the decreased efficiency of BIR, we hypothesized that a fraction of Ade^+^Leu^−^ events in checkpoint deficient mutants might in fact represent not BIR, but GCRs resulting from abnormal stabilization of the broken molecules (similar to discussed in [21], [41]). To detect possible GCRs and to characterize their contribution to heterogeneity of the colonies, we employed PFGE to analyze repair outcomes in 23 individual *rad24*Δ and 11 *rad9*Δ Ade^+/−^ multi-sectored colonies, which comprised Leu^+^ and/or Leu^−^ clones (see, for example, colonies in Fig. 5A, B). In this analysis, we focused on individual colonies with at least one HC sector because they represented the majority of all multi-sectored colonies in both mutants. All Ade^+^Leu^−^ sectors as well as a representative number of Ade^+^Leu^+^ (GC), Ade^−w^Leu^−^ (HC) and Ade^−r^Leu^−^ (CL) sectors from each colony were cloned out and analyzed (see, for example, Fig. 5C for PFGE analysis of all sectors from the colony shown in Fig. 5A). PFGE analyses of colony sectors with Ade^+^Leu^+^, Ade^−w^Leu^−^ and Ade^−r^Leu^−^ phenotypes confirmed that they resulted from GC, HC, and CL, respectively, as predicted (Fig. 5C and see also Fig. 4A, B).

PFGE analysis of the Ade^+^Leu^−^ outcomes from individual colonies revealed three categories of outcomes (Fig. 5D). In the first group, which comprised 64% and 35% of all Ade^+^Leu^−^ events in *rad24*Δ and *rad9*Δ, respectively, the chromosome structure was similar to the one observed in true BIR outcomes. However, the high fraction of HC events in the respective colonies makes it highly likely that many of these Ade^+^Leu^−^ outcomes resulted not from BIR, but from segregation of a HC repair product with an intact copy of the full-length chromosome III (similar to events described in [12], [13]; see Materials and Methods for details). Other Ade^+^Leu^−^ outcomes were represented by events where DSB repair resulted in formation of GCRs. Thus, approximately 13% of Ade^+^Leu^−^ from multi-sectored colonies of each of the checkpoint-deficient mutants were chromosomal rearrangements (called CRs), where the broken recipient chromosome was aberrantly stabilized by *de novo* telomere formation or through ectopic recombination between a Ty or delta element in the *MAT*a-containing chromosome and Ty or delta element located in an ectopic position (Fig. 1F, Fig. 5D; similar to previously demonstrated [21]). These CRs carried an unchanged donor chromosome (a 356 kb band that hybridized to the *ADE3*-specific probe) and a recipient band of any size (different from 346 kb) that hybridized to *ADE1* (Fig. 4A, 4B; CR). Previously, the structure of similar CR events was characterized by array-CGH [21] and it was determined that CRs often result from ectopic BIR initiated by strand invasion of Ty or delta elements of the broken recipient chromosome into Ty or delta elements at ectopic positions.

A significant fraction of Ade^+^Leu^−^ outcomes represented a new type of GCR that contained a single BIR-sized (346 kb) recipient chromosome and a rearranged donor (a band other than 356 kb that hybridized to *ADE3* (Fig. 4A, 4B; half-crossover-initiated cascades (HCC), Fig. 5D)). We posited that these repair outcomes likely arose from the rupture of the donor chromosome during HC formation, resulting in an *ADE3*-containing broken fragment that was stabilized by ectopic recombination. The possibility of such HCC events has been previously discussed [12], but never demonstrated. Here we found that 61% and 73% of *rad24*Δ and *rad9*Δ multi-sectored colonies contained at least one Ade^+^Leu^−^ sector that represented a HCC event (Fig. 5D), the molecular structure of which was further analyzed by array-CGH (see below).

**Figure 6:**
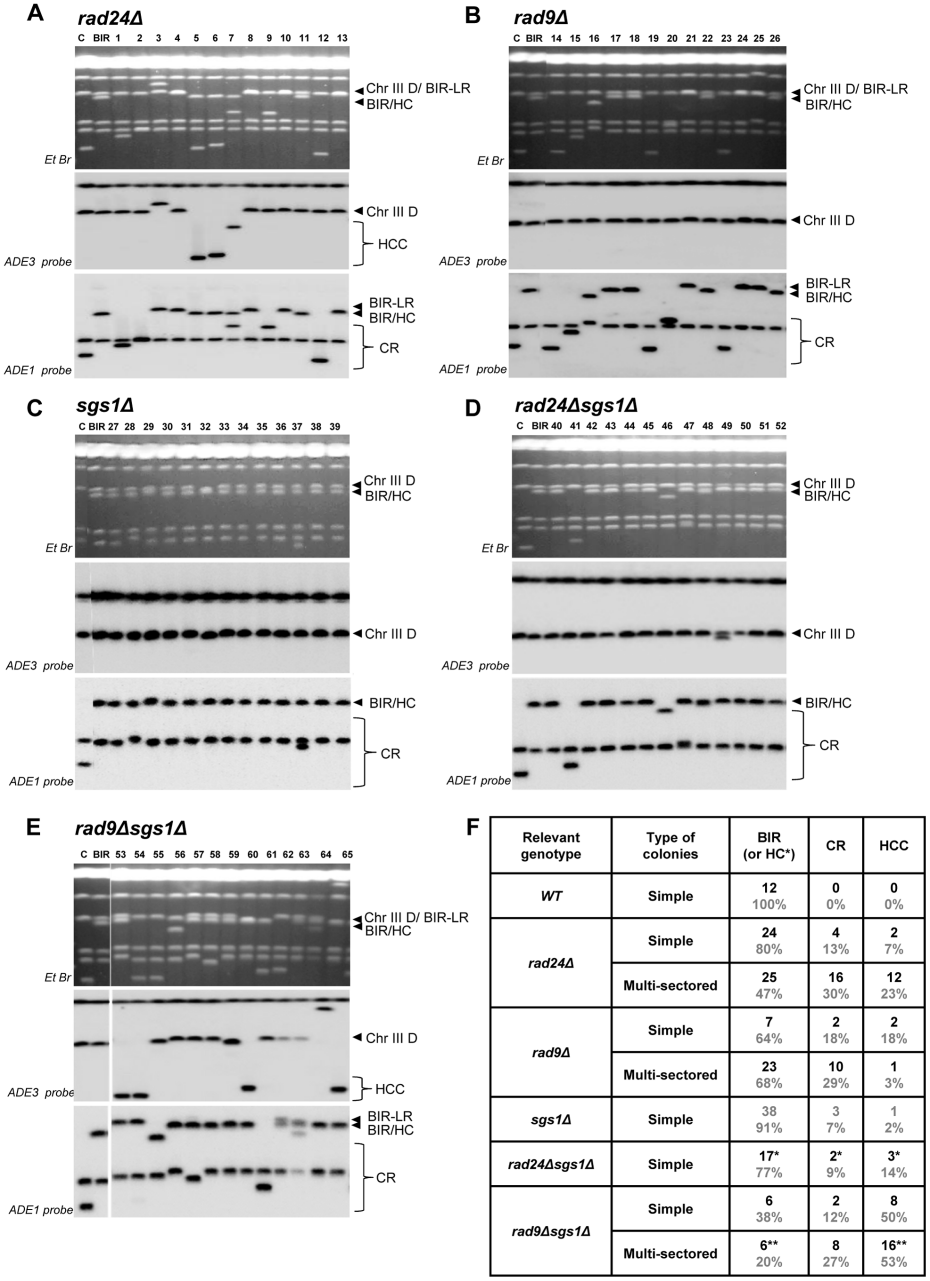
Structural analysis of random Ade^+^Leu^−^ DSB repair outcomes. PFGE analysis of randomly selected Ade^+^Leu^−^ DSB repair events obtained in (A) *rad24*Δ, (B) *rad9*Δ, (C) *sgs1*Δ (D) *rad24Δsgs1*Δ, and (E) *rad9*Δ*sgs1*Δ. On the top of each panel: Ethidium bromide-stained PFGE gel; in the middle and on the bottom: Southern blot analysis of the PFGE gel using *ADE3*- and *ADE1*-specific probes, respectively. Lanes labeled “C”: genomic DNA from *rad24*Δ before DSB induction. Lanes labeled “BIR” depict DNA from a BIR outcome. See Fig. 4A and 4B for the structure of BIR, CR, and HCC repair events. Note that the size of BIR product in BIR-LR outcomes is 356 kb instead of 346 kb because they result from strand invasion centromere proximal to *NAT* (See Materials and Methods for details). For *rad24*Δ (shown in A), *rad9*Δ (shown in B) and *rad9*Δ*sgs1*Δ (shown in E), lanes 1–13, lanes 14–26 and lanes 53–65 represent Ade^+^Leu^−^ sectors from multi-sectored colonies. For *sgs1*Δ (C) and *rad24*Δ*sgs1*Δ (D) lanes 27–39 and 40–52 represent Ade^+^Leu^−^ sectors from simple colonies. (F) The distribution of repair events among random Ade^+^Leu^−^ outcomes in simple and multi-sectored colonies calculated based on analyses presented in A–E. BIR or HC classes represent Ade^+^Leu^−^ outcomes that have chromosome structure similar to BIR (or BIR-LR), but could also represent instances of HC co-segregation with an intact donor chromosome during mitosis (see Results and Materials and Methods for details).

We conclude that premature onset of mitosis resulting from a defective checkpoint leads to aberrant processing of BIR intermediates resulting in frequent HCs and other GCRs. We observed that more than 74% and 91% of all analyzed colonies in *rad24*Δand *rad9*Δ, respectively, contained at least one CR or HCC sector. Also, both HCCs and CR events were frequently observed among Ade^+^Leu^−^ events obtained from unselected (random) colonies in both *rad9*Δ and *rad24*Δ mutants, but were very rare in the wild type strains (Fig. 6A, 6B, 6F, and [9], [12]). In addition, the analysis of strains containing the *pol3-t* mutation, which increased the frequency of HC formation (discussed in the previous section) also revealed DSB-induced HCC outcomes (Fig. S3).

### Analysis of half-crossover-initiated cascades (HCC)

**Figure 7:**
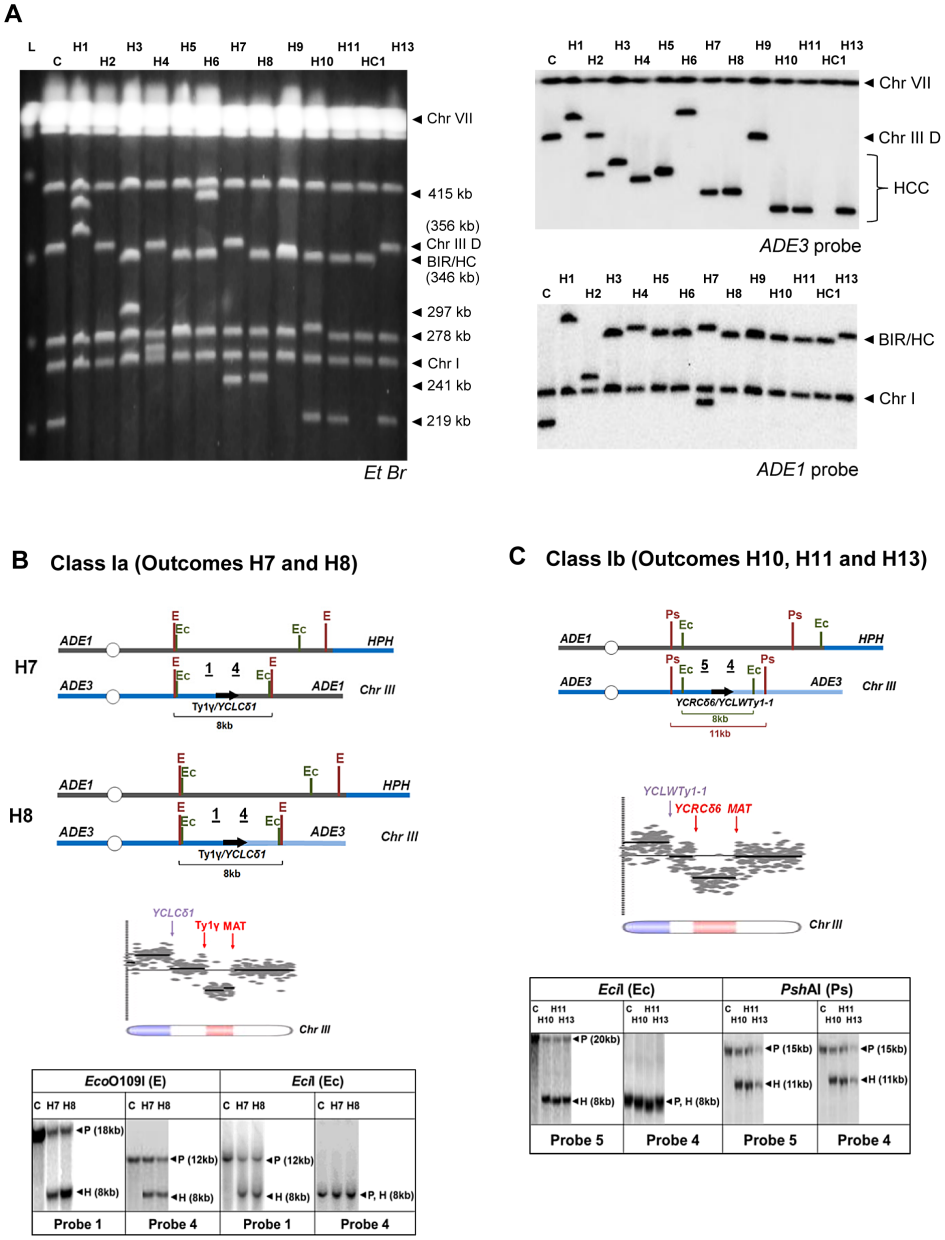
Analysis of HCC outcomes from *rad24* Δ**.** (A) PFGE analysis of HCC outcomes (H1–H11, H13), and one HC outcome (HC1). Left: Ethidium bromide-stained gel; right top and bottom: Southern blot hybridization with *ADE3-* and *ADE1*-specific probes, respectively. (B) The schematic diagram of HCC outcomes H7 and H8 (Class Ia). Array-CGH analysis shows a deletion (red) in Chr. III (between FS1 (Ty1γ; 150235 bp position) and *MAT* (200142 bp position)) and a duplication (blue) of sequences in the same Chr. III (located centromere-distal to *YCLCδ1* (83055 bp position)). Underlined numbers in the schematic diagram (1 and 4) indicate the positions of hybridization probes used for Southern analysis. The positions of *Eco*O109I (E) and *Eci*I (Ec) restriction sites are indicated. The Southern blot shows H7 and H8 digested with *Eco*O109I and hybridized to Probe 1 and Probe 4. As expected, the size of the DNA fragment corresponding to the HCC junction (H) was approximately 8 kb. Similarly, an 8 kb HCC junction (H) fragment was detected when H7 and H8 were digested with *Eci*I and hybridized to Probe 1 and Probe 4. P: the positions of bands corresponding to the original (unrearranged) chromosomes. (C) The schematic diagram of HCC outcomes H10, H11 and H13 (Class Ib) based on the results of array-CGH analysis. Array-CGH analysis shows a deletion (red) in Chr. III (between *YCRCδ6*; 124250 bp position) and *MAT* (200142 bp position)) and a duplication (blue) of sequences in the same Chr. III (located centromere-distal to *YCLWTy1-1*, which corresponds to *YCLWδ15* in SGD (83110 bp position)). Underlined numbers in the schematic diagram (4 and 5) indicate the positions of probes used for Southern hybridization. The positions of *Eci*I (Ec) and *Psh*AI (Ps) restriction sites are indicated. As expected from the maps, the size of the DNA fragment corresponding to the HCC junction (H) was approximately 8 kb following digestion with *EciI* and 11 kb following digestion with *Psh*AI.

As indicated above, the majority of multi-sectored colonies in *rad9*Δ and *rad24*Δ mutants contained at least one HCC event characterized by the presence of a 346 kb band that hybridized to *ADE1*, as well as a second band of varying size (other than 356 kb) that hybridized to an *ADE3*-specific probe and represented a GCR that resulted from breakage and stabilization of the donor chromosome (Fig. 4B; HCC). We used comparative genomic hybridization (array-CGH) to further characterize the nature of 13 stabilized donor chromosomes obtained from HCC events identified in *rad24*Δ mutants. (Fig. 7A) Based on array-CGH, the stabilized donor chromosomes resulting from HCCs were divided into three main classes that accounted for all 13 analyzed outcomes: isochromosomes (Class I), translocations (Class II), and secondary BIR events (Class III) (Table S2).

Class I rearrangements included 9 of the 13 HCC events analyzed by array-CGH (Fig. 7A, Table S2). Each of these events had in common a deletion of sequences in the right arm of chromosome III and a duplication of sequences from the opposite chromosome arm (Fig. 7B, 7C; Table S2), thus forming an isochromosome. (Class I is subdivided into Class Ia, Ib, Ic, and Id depending on the point of recombination and other details of the process; see Table S2). We propose that the formation of outcomes Ia, Ib, and Ic was initiated by invasion of the broken recipient into the full donor chromosome III, which led to the formation of an HC represented by a 346 kb band hybridized to *ADE1-*specific probe (Fig. S4, S5). The resulting broken *ADE3-*containing fragment was then resected and subsequently repaired by non-allelic homologous recombination between a Ty or delta element located in the right arm of chromosome III and a Ty or delta element located in the left arm of chromosome III. For example, in the case of Class Ia outcomes (H7 and H8), the recombination occurred between the Ty1γ element in FS1 and a delta element *YCLCδ1* (Fig. 7A, Table S2, Fig. S4). The predicted size of such an isochromosome (calculated based on [19], [42], and also based on the data from SGD) was approximately 245 kb (Table S2), which was consistent with the size of the *ADE3*-hybridizing band observed by PFGE analysis (Fig. 7A). Our proposed molecular structure was further confirmed through a detailed Southern analysis using the restriction enzymes *Eco*O1091 and *Eci*I and Probe 1 (FS1-specific) and Probe 4 (specific to the region of chromosome III located centromere-distal to *YCLCδ1* (Fig. 7B, Fig. S4, Table S3). Analogously, in the case of Class Ib outcomes (H10, H11, H12 and H13), recombination occurred between delta elements *YCRCδ6* and *YCLWTy1-1* [19], which corresponds to *YCLWδ15in SGD*. The predicted size of such an isochromosome was approximately 219 kb (Table S2), which was consistent with the size of the *ADE3*-hybridizing band observed by PFGE analysis (Fig. 7A). The structures of H10, H11, and H13 were further confirmed by Southern analysis using the restriction enzymes *Eci*I and *Psh*AI and also Probe 4 and Probe 5 (specific to region of chromosome III located centromere proximal to *YCRCδ6*) (Fig. 7C, Fig. S5, Table S3). Also see Text S1 and Fig. S6 for a detailed description of Class Ic HCC outcomes (H4 and H5). The formation of the HCC outcome H2 (Class Id) can be explained similarly to other Class I events, but likely involved two half-crossover events (see Text S1 and Fig. S7).

Class II included only one of the 13 HCC outcomes, outcome H3. H3 was determined to result from a deletion in chromosome III between positions of FS1 (a tandem repeat of Ty1 elements) and *MAT*, and a duplication of all sequences located on chromosome V distal to a solo delta element *YERCdelta16* (Fig. S8 and Table S2). We propose that the formation of H3 was initiated by HC formation between the left arm of the recipient and the right arm of the donor chromosomes (similar to Class I), which resulted in a broken *ADE3-*containing fragment. This fragment was subsequently repaired by recombination between a Ty element in FS1 and a delta element located in the right arm of chromosome V, which led to the formation of translocation. (See Text S1, Table S2 and Fig. S8 for the details of structural analysis of H3).

Class III rearrangements included 3 of the 13 analyzed HCC outcomes and were further divided into IIIa and IIIb. Class IIIa was represented by the outcome H9. Array-CGH analysis of this outcome indicated a duplication of chromosome III sequences from *MAT* through the telomere indicative of BIR repair; however, PFGE analysis revealed that both *ADE1* and *ADE3*-hybridizing chromosomes were equal in size (346 kb long; Fig. S9, Table S2). We hypothesized that, similar to other HCC events, the formation of H9 was initiated by HC, which led to the formation of an *ADE3*-containing broken fragment. This broken donor fragment was stabilized through invasion into the HC product centromere proximal to *NAT* followed by BIR that copied the right arm of HC (Fig. S8). Therefore, we named this outcome a “secondary BIR event”. Importantly, 8 of 24 HCCs that were originally identified by PFGE showed a pattern similar to H9 (both *ADE1*- and *ADE3*-hybridizing bands were approximately 346 kb; data not shown), which suggests that all of them most likely represented secondary BIR events, even though only H9 was analyzed by array-CGH. Therefore, it appears that secondary BIR events are relatively common among BIR outcomes in checkpoint-deficient mutants.

The array-CGH analysis of the outcomes H6 and H1 (class IIIb) also showed a duplication of chromosome III sequences from *MAT* through the telomere indicative of BIR, which made them similar to secondary BIR events. However, these events included additional rearrangements (Table S2; see also Text S1 for details).

Overall, we conclude that interruptions during BIR repair in checkpoint-deficient mutants lead to frequent breakage of the donor chromosome that results in further cascades of DNA instabilities.

### *SGS1* modulates distribution of repair outcomes in checkpoint-deficient mutants

It has been demonstrated that the absence of Rad9 increases the rate of resection at a DSB, which could contribute to the increased frequency of chromosome loss and GCRs we observed in *rad9*Δ mutants [43], [44]. Therefore, we tested whether *sgs1*Δ and *rad50*Δ known to decrease the efficiency of DSB resection) [45]–[47] affected the distribution of repair outcomes in *rad9*Δ and *rad24Δ*.

We observed that deletion of *SGS1*, which is required for long-range 5′-strand resection, in *rad24*Δ, dramatically reduced the frequency of chromosome loss (P<0.0001) (Fig. 4E), and virtually eliminated all multi-sectored colonies (Fig. 4C). The majority of colonies formed in *rad24*Δ*sgs1*Δ were fully Ade^+^Leu^−^, and their PFGE analysis indicated that they contained normal BIR events (Fig. 4E; Fig. 6D, 6F), even though they can also represent secondary BIR events. Deletion of *SGS1* in *rad9*Δ also affected the distribution of repair outcomes. Compared to *rad9*Δalone, the frequency of chromosome loss was decreased (P<0.0001), while HCC were increased in the double mutant (P<0.0001) (Fig. 4E; Fig. 6E, 6F). In addition, we observed that deletion of *RAD50*, which is involved in end processing near the DSB site [45], did not affect the distribution of repair outcomes in *rad9*Δ or *rad24*Δ (Fig. 4E). Importantly, the deletion of *SGS1* gene alone (in strains with functional checkpoint response) led to only a modest change in distribution of DSB repair outcomes (Fig. 4C, 4E, 6C, 6F.). Overall, our data suggest that deletion of *SGS1* significantly affects the distribution of repair outcomes in the absence of a functional checkpoint response.

## Discussion

### Decreased quality of BIR DNA synthesis promotes HC formation

**Figure 8:**
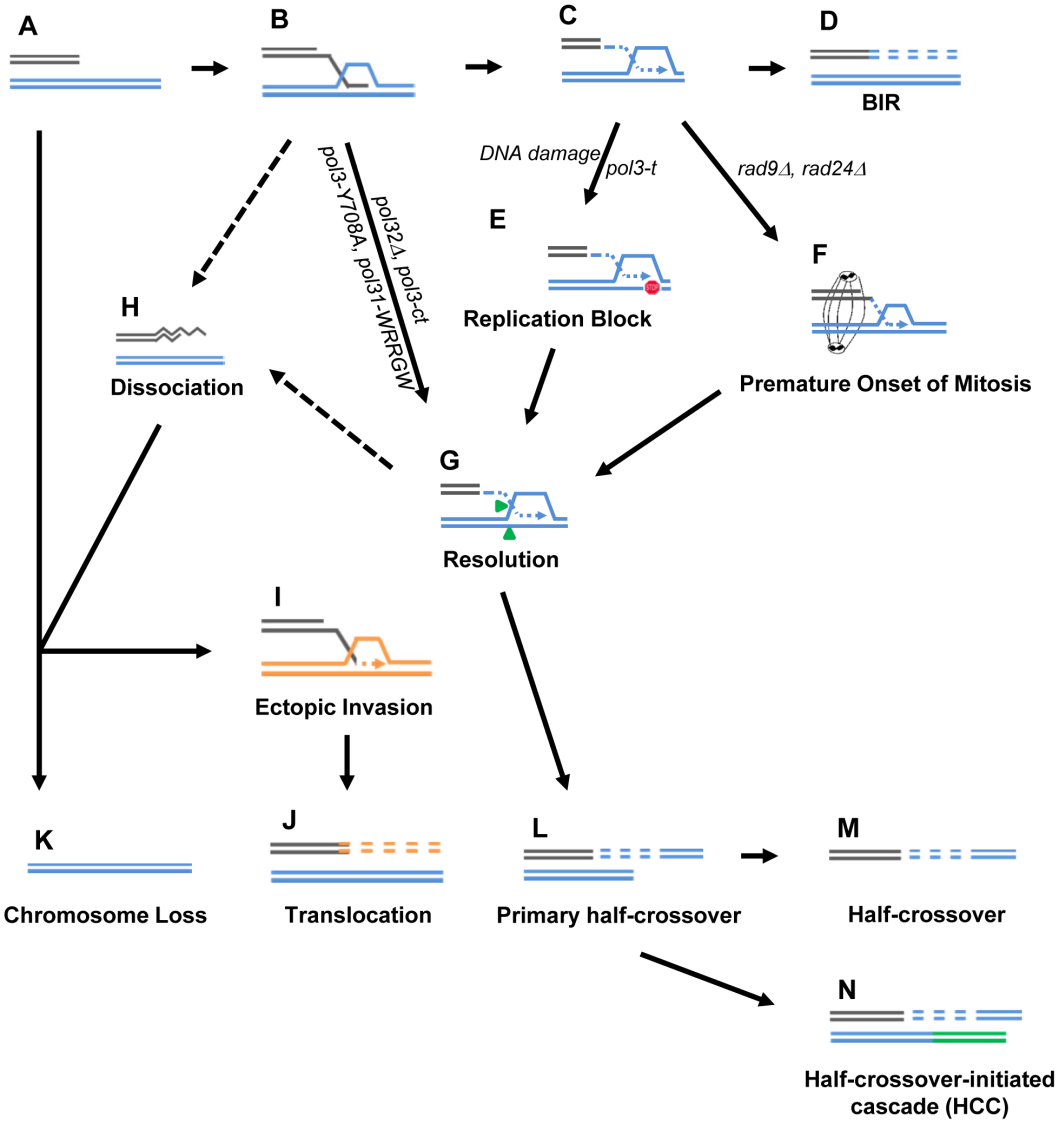
Model for BIR-induced genetic instabilities. (A) One-ended DSB. (B) Invasion of 3′-ssDNA end into a homologous chromosome. (C) Unidirectional DNA synthesis. (D) BIR product. (E) A pause during BIR replication (indicated by the red “stop” symbol) promotes resolution of the Holliday junction (G) and leads to formation of primary half-crossovers (L). (F) Premature onset of mitosis during BIR. (H) Dissociation of a newly synthesized strand from its template during BIR can lead to its invasion at ectopic position (I) resulting in translocation (J). (K) Chromosome loss. (M) Half-crossover. (N) Half-crossover-initiated cascade (HCC).

BIR is a critical mechanism to repair broken chromosomes. Normally, BIR is initiated by a DSB produced in such a way that only one end of the broken molecule is available for repair (Fig. 8A). It thus initiates with a single invasion into a homologous template (Fig. 8B) followed by initiation of DNA synthesis (Fig. 8C) that proceeds to the telomere (Fig. 8D). Increased HC formation and chromosome loss was previously demonstrated in *pol32Δ* and *pol3-ct* mutants during BIR repair where strand invasion was successful, but DNA synthesis could not be (or was poorly) initiated [12], [13]. Likewise, here we report a similar phenotype in strains containing other mutations in Polδ, *pol3-Y708A* (a mutation affecting the catalytic subunit of Polδ) [31] and *pol31-WRRGW* (a mutation in the Pol31 subunit of Polδ) [32]. For each of these cases, we propose that HC formation results from resolution of HJ structures that persist when BIR DNA synthesis is not initiated (Fig. 8B, 8L). It has been suggested that Mus81 resolves HJs and therefore may contribute to HC formation [13].

We demonstrate that BIR interrupted at various stages of its progression, for example during replication, also stimulates HCs. This most likely occurs in *pol3-t* mutants because the processivity of Polδ is compromised (Fig. 8C, 8L). Similarly, results from another recent study [15] demonstrate that the deletion of *PIF1*, which encodes a DNA helicase specifically required for DNA synthesis during BIR [48], also leads to more frequent HCs. We speculate that in these mutants, DNA synthesis is successfully initiated but proceeds with frequent stops, thereby promoting HC formation. We postulate that a similar mechanism of paused DNA synthesis can explain the increase in HC formation we observed in wild type cells exposed to the DNA damaging agents (MMS or 4-NQO) during BIR repair (Fig. 8C, 8L). Regardless of the mechanism that leads to paused replication, these data support our hypothesis that interruptions in DNA replication during BIR induce HCs. Interestingly, mutations affecting Polε that were investigated so far did not promote HCs. This might be explained by limited participation of Polε in BIR [11]. Curiously, the mutation in Polα (*pol1-1*) led to decreased HCs compared to wild type, which may indicate that *pol1-1* delays accumulation of BIR intermediates that are resolved to produce HCs.

We previously demonstrated that successful completion of BIR replication requires checkpoint machinery to maintain cell cycle arrest until repair is completed [9]. Consistently, here we observed that the premature onset of mitosis in checkpoint-deficient cells undergoing BIR repair led to an increased frequency of HCs (Fig. 8F, 8L). Formation of HC molecules could result from a signal from the cell to resolve the HJ structure as previously discussed, but mechanical rupture of BIR intermediates initiated by chromosomal segregation is also a possibility. In checkpoint-deficient mutants, we also frequently observed chromosome loss and translocations, which we propose result from strand dissociation that can be stimulated by HJ resolution (Fig. 8G, 8H). 5′-to-3′ DNA resection following dissociation may lead to chromosome loss (Fig. 8K) or to ectopic strand invasion at positions of DNA repeats (Fig. 8I) resulting in translocations (Fig. 8J). Alternatively, elevated chromosome loss and translocations may result from increased 5′-to-3′ resection of DSB ends prior to strand invasion (Fig. 8A, 8K) or following unwinding of a D-loop (Fig. 8B, 8H)

### Half-crossovers initiate cascades of genetic instability

**Figure 9:**
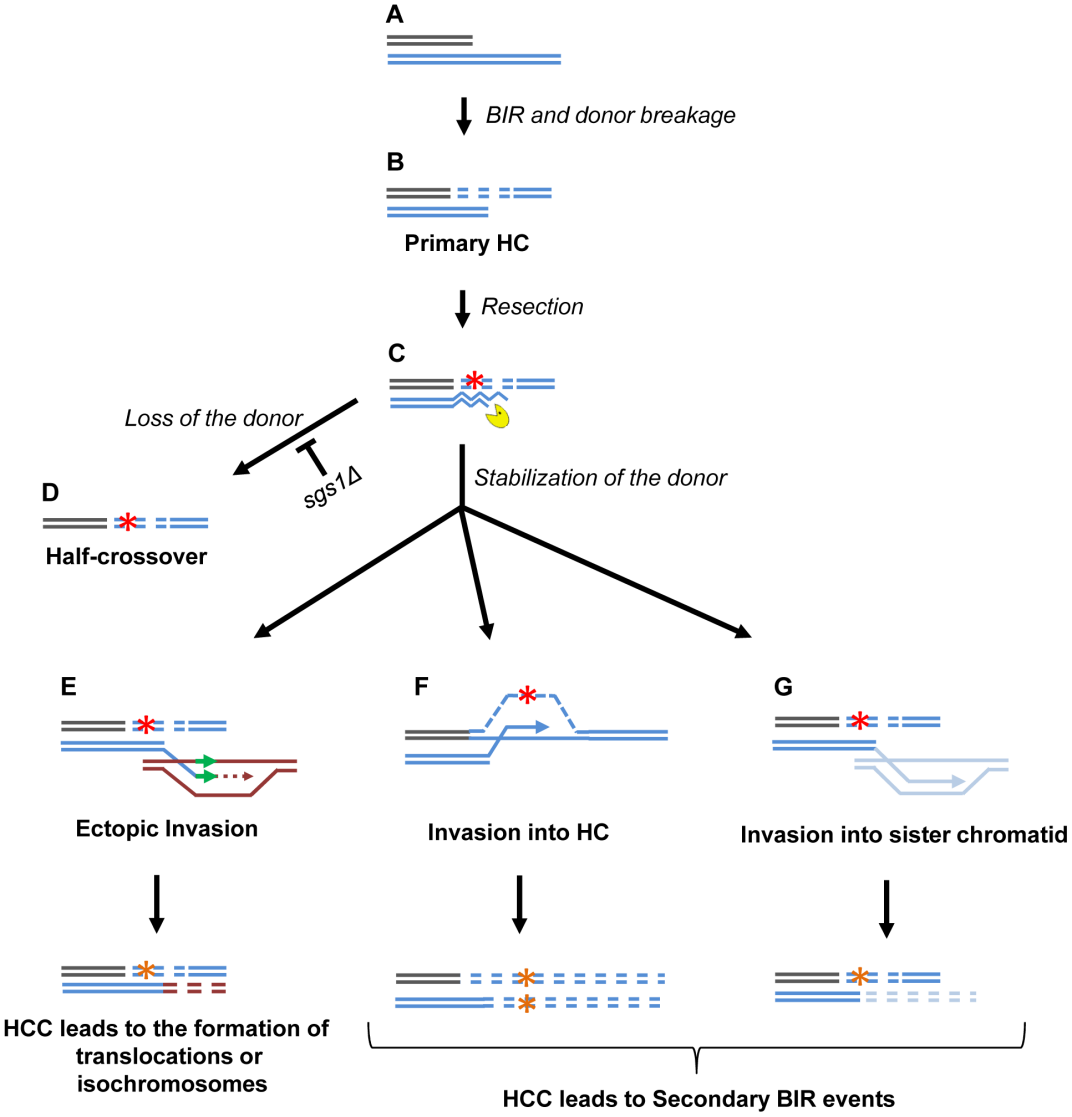
Model of half-crossover-initiated cascades (HCC). (A) One-ended DSB. (B) Formation of half-crossover (HC) leading to breakage of the donor chromosome. (C) Resection of the broken donor chromosome. (D) HC associated with the loss of a donor chromosome. (E) Stabilization of the donor chromosome by invasion at ectopic chromosomal location leads to formation of a translocation or of an isochromosome. (F) Stabilization of the donor chromosome by invasion into the initial HC followed by BIR (secondary BIR event). (G) Stabilization of the broken donor by strand invasion into sister chromatid followed by BIR (secondary BIR event). Red stars represent mutagenic errors during DNA synthesis leading to HC formation. Orange stars represent mutations. Secondary BIR in (F) may lead to formation of homozygous mutations (see the text for details).

An important outcome of this study is the discovery of HC-induced cascades (HCC). The existence of HCCs has been previously hypothesized [12], but, until now, had not been demonstrated. HCCs represent DSB repair outcomes that contain a HC product along with a rearranged donor chromosome (Fig. 8N). We propose that HCCs are initiated by a single HC that leads to breakage of the donor chromosome (Fig. 9A,B). The new DSB in the donor molecule undergoes 5′ to 3′ resection (Fig. 9C), and the resulting 3′ DNA end invades a homologous DNA molecule at an ectopic position (Fig. 9E) in the newly formed HC (Fig. 9F), or in the sister chromatid (Fig. 9G). This initiates recombination and can stabilize the broken donor chromosome if repair proceeds through BIR; conversely, this intermediate may also result in HC formation, thereby continuing the cascade of genetic instability. Even when the donor fragment successfully stabilizes through BIR, if an ectopic site such as a Ty or delta element is used for recombination, translocations will occur (Fig. 9E; see also Figs. S4, S5, S6, S7, S8).

In our system, repair of the broken donor chromosome often proceeded by BIR using the recently formed HC (Fig. 9F) or the sister chromatid (Fig. 9G) as a template. We termed these events “secondary BIR”. Among all HCC events analyzed by PFGE, approximately half showed a pattern suggestive of HCC resulting in invasion of the broken donor into the initial HC, even though only one of these cases was analyzed by array-CGH (case H9; Fig. S9). Additionally, 15% of the HCCs analyzed by CGH could be explained by secondary BIR associated with complex rearrangements (cases H1 and H6). It should be noted that all identified secondary BIR events were initiated by strand invasion that occurred centromere proximal to FS2 which resulted in a change in the size of the donor and therefore allowed the detection of these events. It remains possible; however, that many additional secondary BIR events are initiated by strand invasion between *FS2* and *MAT*. This is expected to result in chromosome structures and phenotypes indistinguishable from classic BIR. Therefore, we propose that the actual frequency of secondary BIR events maybe higher than currently estimated. This is significant because secondary BIR events could be more deleterious than classic BIR events. For example, we speculate that secondary BIR may result in homozygous mutations that result when a mutation occurs during DNA synthesis associated with HC formation (Fig. 9F) and is then copied during the repair of the broken donor using the initial HC as a template. Homozygous mutations could be more deleterious than heterozygous ones (reported in association with classic BIR [18]) because they can lead to the manifestation of recessive phenotypes including those leading to cancer.

A significant finding was the formation of multi-sectored colonies consisting of broad genotypic variations by checkpoint-deficient mutants. More than 70% of the multi-sectored colonies contained at least one sector with chromosomal rearrangements of recipient or donor chromosomes, with HCC being a major class of these rearrangements. In addition, we observed that deletion of *SGS1*, which is known to reduce long-range DSB resection, led to a significant increase in HCC frequency in *rad9Δ* mutants. Possibly, reduced resection stabilizes the broken donor chromosome, thus giving it more chances to repair by invading a homologous template (Fig. 9E–G). Alternatively, it is possible that faster initiation and/or progression of BIR that was previously documented in the absence of Sgs1 [7] contributes to the increased stabilization of the donor chromosome and therefore to the increased level of HCC. Interestingly, in *rad24Δsgs1Δ*, the multiple sectoring of colonies was completely eliminated, and the majority of outcomes were indistinguishable from normal BIR. We propose that these events are likely to be secondary HCC resulting from secondary BIR.

In addition to being frequently observed when BIR is induced in checkpoint-defective mutants, HCCs occurred in cases of compromised BIR in polymerase-deficient mutants (*pol3-t*) and when BIR proceeds in the presence of MMS (Sakofsky et al, manuscript submitted). HCCs were also observed in BIR-defective *pif1Δ* mutants [15]. Overall, we propose that ongoing cycles of genetic instability are a ubiquitous outcome of HC formation.

### HC-induced cascades: Potential for promoting genetic instability in humans

We propose that HCs and HCCs may be a mechanism for genetic destabilization leading to various diseases in humans. In particular, we propose HCCs to be a mechanism capable of producing non-reciprocal translocations (NRTs) that have been described in mammalian tumor cells. NRT is a pathway of telomere acquisition by broken chromosomes that results in the donor molecule losing genetic information, including its telomere, and becoming unstable [30]. This destabilization of the donor makes NRTs especially devastating because the events are self-perpetuating and result in cascades of genomic destabilization, including chromosome loss and multiple rearrangements. We propose that the cycles of NRTs can be explained by initiation of BIR followed by its interruption leading to HCCs in tumor cells. Importantly, we suggest that initiation of HCC can be facilitated by checkpoint deficiency, which is frequent in cancer cells. [49], [50]. In addition, our data suggest that cycles of HCCs could also contribute to clonal variations in pre-cancerous cells. Finally, our observation of increased HCs and HCCs resulting from the exposure of cells undergoing BIR to DNA damaging agents, could be of significant relevance to current cancer therapeutic strategies where anti-replication drugs are used in combination with agents that induce DSBs (for example, X-ray and gamma irradiation). We propose that DSBs induced by these agents may initiate BIR, which will frequently lead to NRT events in the presence of cancer drugs that inhibit replication. Because NRT events often initiate cascades of genetic instability, promoting such events in cancer cells could be one mechanism of rapid GCR formation that could result in negative oncotherapy outcomes such as secondary tumorigenesis and drug resistance.

## Materials and Methods

### Yeast strains

All yeast strains (Table S1) were isogenic to AM1003 [12] which is a chromosome III disome with the following genotype: *hmlΔ::ADE1/hmlΔ::ADE3 MAT*a-*LEU2*-tel/*MATα-inc hmrΔ::HPH* FS2Δ::*NAT*/FS2 *leu2/leu2-3*,*112 thr4 ura3-52 ade3::GAL::HO ade1 met13*. In this strain, the *HO* endonuclease-induced DSBs introduced at *MAT*a are predominantly repaired by BIR because the portion of the chromosome centromere-distal to *MAT*a is truncated to leave only 46 bp of homology with the donor sequence [9], [12]. The majority of single-gene deletion mutants were constructed by transformation with a PCR-derived *KAN-MX* module flanked by terminal sequences homologous to the sequences flanking the open reading frame of each gene [51]. The resulting constructs were confirmed by PCR and by phenotype. *sgs1*Δ mutants were constructed by PCR-amplification of *sgs1*Δ*URA3* from the strain yWH239 [48] using oligonucleotides complementary to sequences flanking *SGS1*. To disrupt *RAD50*, plasmid pNKY83 [52] was digested simultaneously with *Bgl*II and *Eco*RI and transformed into recipient strains that were subsequently screened for a Ura^+^ phenotype (*rad50::hisG::URA3::hisG*).

Several polymerase-deficient mutants were constructed using the “pop-in-pop-out” method. The “pop-in” step involved transformation of strain AM1003 with the following plasmids: 1) *Hpa*I-linearized p170-Y708 containing the *pol3-Y708* allele [31], 2) *Age*I-linearized p173-Y831 containing the *pol2-Y831* allele [31], 3) *Sac*I-linearized pCM54 containing the *pol1-1* allele [36], 4) *Mfe*I-linearized pRS306-pol31-WRRGW containing the *pol31-WRRGW* allele [32], 5) *Hpa*I-linearized p171 containing the *pol3-t* allele [34]; and 6) *Eco*RI-linearized p2A5 containing the *pol2-1* allele [35]. In every case, the “pop-in” step was followed by a “pop-out” step that involved growth of transformants on rich medium for two days followed by selection of Ura^−^ outcomes on 5-FOA.

### Media and growth conditions

Rich medium (yeast extract-peptone-dextrose [YEPD]) and synthetic complete medium, with bases and amino acids omitted as specified, were made as described [53]. YEP-lactate (YEP-Lac) and YEP-galactose (YEP-Gal) media contained 1% yeast extract and 2% Bacto peptone supplemented with 3.7% lactic acid (pH 5.5) or 2% (w/v) galactose, respectively. Yeast cultures were grown at 30°C or at 20°C (in the case of yeast strains bearing polymerase mutations, which rendered them temperature-sensitive). As indicated, MMS or 4-NQO (Sigma Aldrich) was added to rich medium for some experiments.

### Kinetics of DSB repair

The kinetics of DSB repair was examined in time-course experiments as described previously [12]. YEP-Lac (500 to 1000 ml was inoculated with approximately 2×10^6^ cells/ml. Cultures were grown at 30°C overnight to reach a concentration of approximately 5×10^6^ cells/ml. *HO* endonuclease was induced by the addition of galactose to achieve a final concentration of 2%. For PFGE gel electrophoresis, 50 ml aliquots were removed, and sodium azide was added to achieve a concentration of 0.1% to stop DNA repair processes. Extraction of DNA embedded in 0.55% agarose plugs was performed as described [9]. For fluorescence-activated cell sorter (FACS) analyses, 5 ml aliquots were removed, cells were spun, diluted, fixed by the addition of 70% ethanol, and stored at 4°C. FACS analysis was performed using propidium iodide with a Becton Dickinson fluorescence-activated cell analyzer, similar to [9].

PFGE was performed by running genomic DNA embedded in agarose plugs at 6 V/cm, for 40 hours (initial switch time 10 s; final switch time 35 s) followed by Southern blotting and hybridization using ^32^P- labeled DNA probes containing either an *ADE1* (*Sal*I fragment from pJH879) (similar to [9], [12]) or *ADE3* (obtained by PCR amplification of chromosome VII from 907979–908735 bp) sequence. The images were analyzed using GE Healthcare Typhoon FLA 9500. The kinetics of accumulation of BIR product was measured using an *ADE1*-specific fragment as a probe. To account for variation in DNA loads, intensities of the bands corresponding to the intact chromosome III, as well as to the repaired chromosome III, were normalized to intensities of the bands corresponding to chromosome I, which also hybridizes to the *ADE1*-specific probe. The efficiency of BIR repair, presented as the percentage of truncated chromosome III that was converted to BIR product, was calculated by dividing the normalized intensity of a repair band by the normalized intensity of uncut, truncated chromosome III. Results of three time-course experiments were used to calculate the average ± SD BIR efficiency for each strain. Cell viability following exposure to the DNA damaging agents was determined as a ratio of the number of colony forming units (CFU) observed experimentally and the number that was predicted based on the cell concentration determined using hemocytometer prior to plating. The viability of cells following treatment with DNA damaging agents was as follows: for DSB repair in the presence of MMS: (20±7)% and (14±7)% for 2.4 mM and 6 mM MMS respectively; for DSB repair in the presence of 5.3 µM 4-NQO: (10±5)%; following MMS treatment without DSB: (23±8)% and (8±3)% for 2.4 mM and 6 mM MMS respectively; and following 5.3 µM 4-NQO treatment without DSB: (19±4)%.

### Analyses of distribution of DSB repair events

To monitor the repair of HO-induced DSBs in individual colonies, we harvested logarithmically growing cells grown in YEP-Lac at 30°C and plated them on YEP-Gal. The resulting colonies were then replica plated onto omission media to examine the *ADE1*, *ADE3*, *LEU2*, and *NAT* markers. When temperature-sensitive strains bearing *pol3-t* or *pol1-1* polymerase mutations were used, the cells were grown in YEP-Lac at 20°C. Following plating on YEP-Gal, the cells were incubated at 30°C for 24 hours (a length of time sufficient to complete BIR), and then incubated at 20°C until the colonies were full-grown. To test the effect of DNA damage, the cells were grown to log phase in YEP-Lac medium at 30°C, incubated in galactose-containing media for 30 minutes (to induce DSB), and then incubated with or without a DNA damaging agent (MMS or 4-NQO) for 7 hours. (The time for incubation with DNA damaging agents was selected based on the known kinetics of BIR [12]). The DNA damaging agents were then deactivated by treatment with 10% sodium thiosulfate prior to serial dilution and plating of cells onto YEPD. Repair events were identified by a phenotypic analysis after replica plating onto omission media, and also by PFGE. Gene conversion (GC) outcomes displayed an Ade^+^Leu^+^ phenotype and contained two copies of chromosome III: a 356 kb chromosome that hybridized to an *ADE3*-specific probe and a short (217 kb) chromosome that hybridized to an *ADE1*-specific probe (Fig. 1C). The absence of repair led to CL, which was detected by formation of Ade^−r^Leu^−^ colonies containing a single, 356 kb chromosome III, which hybridized to the *ADE3*-specific probe (Fig. 1D). Formation of Ade^−w^Leu^−^ colonies or colony sectors indicated formation of HCs. These cells contained a single, 346 kb chromosome III that hybridized to the *ADE1*-specific probe (Fig. 1E). Ade^+^Leu^−^ phenotypes could result from several repair outcomes: BIR, HC (when it co-segregates with an intact copy of the donor chromosome during mitosis [12]), from CRs [21] or from HCC events. CRs and HCC events were identified by PFGE. CRs carried a 356 kb band that hybridized to the *ADE3*-specific probe and a band of any size (different from 346 kb) that hybridized to *ADE1* (Fig. 1F). HCC contained a single, 346 kb band that hybridized to an *ADE1*-specific probe in addition to a band of varying size that hybridized to *ADE3* (Fig. 1G). BIR usually carried a 356 kb band that hybridized to *ADE3* and a 346 kb band that hybridized to *ADE1* (Fig. 1B). Occasionally, BIR resulted from long 5′-3′ resection beyond *NAT*, followed by strand invasion that occurred centromere proximal to *NAT*. This led to the formation of BIR outcomes that were Ade^+^Nat^−^Leu^−^ (BIR-LR) with donor and recipient chromosomes of identical (356 kb) sizes (Fig. 5C). PFGE could not distinguish between BIR and events where a HC co-segregated with an intact donor chromosome. Therefore, we assumed the number of Ade^+^Leu^−^ HCs to be equal to the number of Ade^−w^Leu^−^ HCs based on the idea that an HC product should co-segregate with an intact copy of the donor chromosome in half of the cases of HC formation. Overall, the formula to calculate the number of BIR events was as follows: BIR = (number of Ade^+^Leu^−^)−(GCR+HCC+HC).

The distribution of various types of repair among all repair events was determined differently for simple colonies (containing <3 repair sectors) and for multi-sectored colonies (containing ≥3 repair sectors). The frequency among simple colonies was determined as previously described in [9]. The frequency of each repair outcome in multi-sectored colonies was determined as the sum of all sectors belonging to this phenotypic class divided by the total number of sectors analyzed.

Importantly, since yeast strains used in this study did not clump during plating, we proposed that multi-sectored colonies originated from single cells, where DSB repair was preceded by mitotic divisions. This idea was also supported by our experiments where individual checkpoint-deficient cells were micro-manipulated on galactose containing plates, which led to the formation of multi-sectored colonies (data not shown).

In total, the following number of colonies were scored in experiments aimed to determine the effect of defective polymerases on half-crossover formation for each subsequent strain: Pol^+^ (wt) – 1192 colonies; *pol3Y-708A* – 2428 colonies; *pol3-t* – 1240 colonies; *pol31-WRRGW* – 776 colonies; *pol2-Y831A* – 2491 colonies; *pol2-1* – 896 colonies; and *pol1-1* – 287 colonies. In addition, the following number of colonies were scored in experiments aimed to determine the effect of DNA damage on half-crossover formation for each subsequent condition: DSB+no damage: – 2583 colonies; DSB+5.3 µM 4-NQO – 2645 colonies; DSB+2.4 mM MMS – 526 colonies; DSB+6 mM MMS – 1186 colonies; no DSB+5.3 µM 4-NQO – 1629 colonies; no DSB+2.4 mM MMS – 1407 colonies; no DSB+6 mM MMS – 778 colonies; no DSB+no damage - 1072 colonies. The number of colonies scored in experiments aimed to determine the distribution of repair outcomes in checkpoint-deficient mutants was as follows: Rad^+^ (wt) – 718 colonies, *rad24*Δ – 756 colonies; *rad9*Δ – 465 colonies; *rad24*Δ*sgs1*Δ - 339 colonies; *rad9*Δ*sgs1*Δ - 338colonies; and *rad9*Δ*rad50*Δ - 340 colonies. Finally, the number of simple (s) and multiple (m) repair events scored during analysis of the effect of checkpoint- deficient mutants was as follows for each strain background: wild type (wt): 1353 s; *rad24*Δ: 671 s and 1782 m; *rad9*Δ: 473 s and 946 m; *rad24*Δ*sgs1*Δ: 677 s; *rad9*Δ*sgs1*Δ: 450 s and 515 m; *rad9*Δ*rad50*Δ: 508 s and 346 m; *sgs1*Δ: 1504 s and 49 m.

### Array-CGH analysis of individual HCC events

The CGH analysis was conducted as described recently [54]. Briefly, genomic DNA was prepared from the agarose-embedded full length chromosome material. DNA from the parental strain was labeled with dUTP-Cy3 and DNA from the derivative strains carrying genome rearrangements was labeled with dUTP-Cy5. The labeled DNAs were mixed and competitively hybridized to custom Agilent 60-mer oligonucleotide microarrays. The arrays were scanned, the images were analyzed, and the CNV regions were identified using GenePix 6.0 and Nexus Copy Number software, respectively.

### Southern analysis of HCC outcomes

For Southern analysis, genomic DNA of DSB repair outcomes was purified by glass bead/phenol method as described [55], digested with the appropriate restriction enzymes, and the resulting fragments were separated on a 0.8% agarose gel. Southern blotting was carried out by standard procedures using ^32^P-labeled DNA probes that were generated by PCR amplification using 24 bp primers (available as Table S3) and genomic DNA of AM1003 as a template. The locations of these probes on chromosome III are as follows: (1) Probe 1, 148247–148549 bp (*SRD1*(FS1)-specific); (2) Probe 3, 167594–167893 bp (*RHB1-*specific); (3) Probe 4, 82015–82365 bp (*KCC4-*specific); and 4) Probe 5, 123682–123981 bp (*CIT2*-specific). The location of the *YER134C*-specific probe on chromosome V is 436745–437044 bp (Probe 2). For all probes mentioned above, the starting and ending coordinates on the corresponding chromosomes are derived from the *Saccharomyces* Genome Database (SGD).

### Statistical analysis

All mutants were analyzed for their effect on BIR repair in at least three independent plating experiments. Results from these independent experiments were pooled if it was determined that the distributions of all events were statistically similar to each other using a Chi-square test. The effects of individual mutations on DSB repair were determined by comparing the resulting pooled distributions of repair outcomes obtained for mutants to the distribution obtained for the wild-type strain (AM1003) by Chi-square tests. Specifically, to determine the effect of various mutations on the frequency of HC, all repair outcomes were divided into two groups: HC (Ade^w^Leu^−^ outcomes) and others (combining all other groups). Comparison of the distributions between these two classes in specific mutants vs. wild type was used to determine whether a mutation affected the frequency of HC. The effect of mutations on other DSB repair outcomes and the effects of exposure to various DNA damaging agents were determined similarly.

## Supporting Information

Figure S1 The effect of polymerase defects on the distribution of DSB repair outcomes. The fraction (%) of various DSB repair outcomes were determined similarly to [12] following DSB induction in strains containing different mutations affecting Polδ, Polε or Polα. For each strain, the data are based on 3–14 independent experiments. Asterisks indicate a statistically significant change as compared to wild type (Pol^+^) cells. The efficiency of BIR was reduced as compared to wild type in *pol3Y-708A*, *pol3-t*, *pol31-WRRGW*, *pol2-1*, *pol1-1*, *pol32*Δ (P<0.0001) and in *pol2-Y831A* (P = 0.0005)). The frequency of chromosome loss (CL) was increased in *pol3Y-708A*, *pol3-t*, *pol31-WRRGW*, *pol2-1*, *pol2-Y831A*, *and pol32*Δ (P<0.0001). The frequency of half-crossovers (HC) was increased in *pol3Y-708A*, *pol3-t*, *pol31-WRRGW*, *and pol32*Δ (P<0.0001). Note: While this figure shows the fraction of HCs among all DSB repair events, Fig. 2 presents the fraction of colonies that are fully or partially HCs.
(TIF)

Figure S2 The effect of DNA damaging agents on the distribution of DSB repair outcomes. The fraction (%) of various DSB repair outcomes was determined similarly to [12] following DSB induction in AM1003 and its repair in the presence of MMS or 4-NQO. The data are based on 3 to 4 independent experiments for different conditions. A statistically significant difference from the distribution of DSB repair events in the absence of DNA damage is indicated by asterisk. The frequency of HC was significantly increased following exposure to MMS and 4-NQO (P<0.0001). For no-DSB control, the experiments were performed similarly to [12], but without addition of galactose. Asterisks indicate a statistically significant change as compared to the no-damage control. Note: While this figure shows the fraction of HCs among all DSB repair events, Fig. 2 presents the fraction of colonies that are fully or partially HCs.
(TIF)

Figure S3 Structural analysis of Ade^+^Leu^−^ DSB repair outcomes in *pol3-t* mutants. PFGE analysis of Ade^+^Leu^−^ DSB repair events obtained from multi-sectored colonies in *pol3-t* mutants. Ethidium bromide-stained gel (top), Southern blot analysis of PFGE gel using *ADE3*-specific (middle) and *ADE1*-specific (bottom) probes are shown. Lane labeled “C”: no-DSB control. Lane labeled “BIR”: DNA from a known BIR outcome.
(TIF)

Figure S4 Structural analysis of HCC outcomes H7 and H8. (A) Array-CGH analysis of HCC outcomes H7 and H8 (Class Ia) shows a deletion (red) in Chr III (between FS1 (Ty1γ; 150235 bp position) and *MAT* and a duplication (blue) of Chr III sequences located centromere-distal to *YCLCδ1* (83055 bp position)). (B) Molecular mechanism explaining the formation of H7 and H8.
(TIF)

Figure S5 Structural analysis of HCC outcomes H10, H11 and H13. (A) Array-CGH analysis of HCC outcomes H10, H11 and H13 (Class Ib) shows a deletion (red) in Chr III (between *YCRCδ6*; 124250 bp position) and *MAT*; and a duplication (blue) of Chr III sequences located centromere-distal to *YCLWTy1-1*, which corresponds to *YCLWδ15* in SGD (83110 bp position). (B) Molecular mechanism explaining the formation of H10, H11, and H13.
(TIF)

Figure S6 Structural analysis of HCC outcomes H4 and H5. (A) Array-CGH analysis of HCC outcomes H4 and H5 (Class Ic) shows a deletion (red) in Chr III (between FS2; 169419 bp position) and *MAT*; and a duplication (blue) of Chr III sequences located centromere-distal to *YCLWTy1-1*, which corresponds to *YCLWδ15* in SGD (83110 bp position). (B) Molecular mechanism explaining the formation of H4 and H5.
(TIF)

Figure S7 Structural analysis of HCC outcome H2. (A) Array-CGH analysis of HCC outcome H2 (Class Id) shows a deletion (red) in Chr III (between FS2; 169419 bp position) and *MAT*; and a duplication (blue) of Chr III sequences located centromere-distal to *YCLWTy1-1*, which corresponds to *YCLWδ15* in SGD (83110 bp position). (B) Molecular mechanism explaining the formation of H2.
(TIF)

Figure S8 Structural analysis of HCC outcome H3. (A) Array-CGH analysis of HCC outcome H3 (Class II) shows a deletion (red) in Chr III (between FS1 (Ty1γ; 149482 bp position) and *MAT*; and a duplication (blue) of sequences in Chr V (located centromere-distal to a delta element located close to *YERCδ16* (435946 bp position)). Underlined numbers (1 and 2) indicate the positions of probes used for Southern hybridization. The positions of *Psy*I (Ps) and *Ppu*MI (P) restriction sites are indicated. The Southern blot of H3 genomic DNA digested with *Psy*I and hybridized to Probes 1 and 2 shows a 12-kb-long DNA fragment at the HCC junction. Also, an 8 kb DNA fragment was obtained when H3 was digested with *Ppu*MI and hybridized with Probe 1 and Probe 2. (B) Model explaining molecular mechanism of H3 formation.
(TIF)

Figure S9 Structural analysis of HCC outcome H9. (A) Array-CGH analysis of HCC outcome H9 (Class IIIa) shows duplication (blue) of Chr III from *MAT* through the telomeric end. (B) Molecular mechanism explaining the formation of H9.
(TIF)

Table S1 Strain list. The list of strains of yeast *Saccharomyces cerevisiae* that were used in this study.
(TIF)

Table S2 The analysis of HCC outcomes in *rad24*Δ cells. Catalog of half-crossover-initiated cascade DSB repair outcomes that were identified in *rad24*Δ and characterized by array-CGH and PFGE. BFB: breakage-fusion-bridge cycle.
(TIF)

Table S3 The list of primers used to prepare hybridization probes. The 5′-3′ sequences of primers use to prepare hybridization probes by PCR are presented.
(TIF)

Text S1 Supplemental Results and Discussion. Description of HCC outcomes that were identified in *rad24*Δ and characterized by array-CGH and PFGE. The discussion of the mechanisms of their formation.
(DOCX)
