# Supplementary figures and images for: Cascades of Genetic Instability Resulting from Compromised Break-Induced Replication
Source: PLoS Genet. 2014 Feb 27;10(2):e1004119. doi: 10.1371/journal.pgen.1004119 (PMC3937135; doi:10.1371/journal.pgen.1004119)

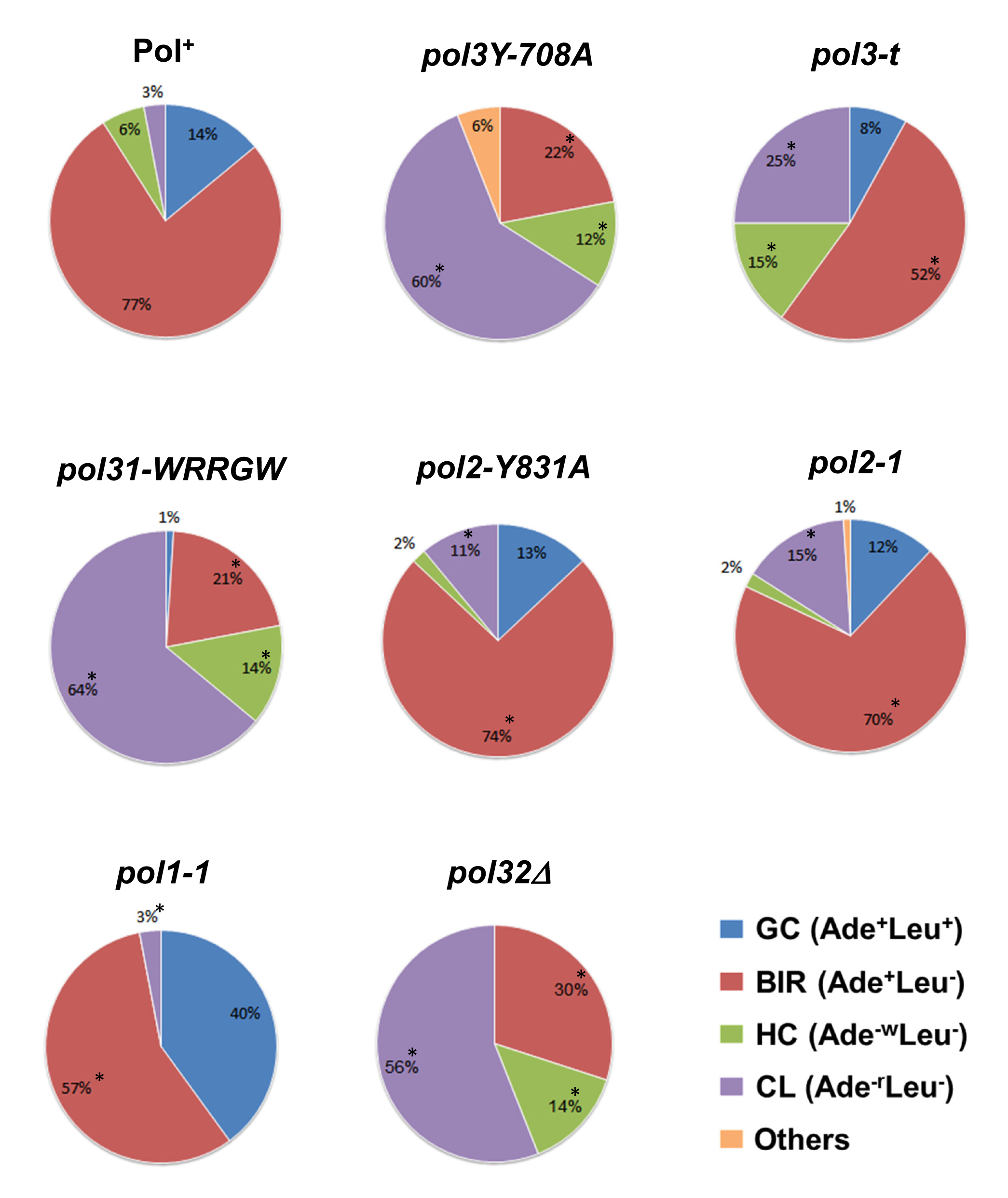

Supplement: Figure S1 — The effect of polymerase defects on the distribution of DSB repair outcomes. The fraction (%) of various DSB repair outcomes were determined similarly to [12] following DSB induction in strains containing different mutations affecting Polδ, Polε or Polα. For each strain, the data are based on 3–14 independent experiments. Asterisks indicate a statistically significant change as compared to wild type (Pol+) cells. The efficiency of BIR was reduced as compared to wild type in pol3Y-708A, pol3-t, pol31-WRRGW, pol2-1, pol1-1, pol32Δ (P<0.0001) and in pol2-Y831A (P = 0.0005)). The frequency of chromosome loss (CL) was increased in pol3Y-708A, pol3-t, pol31-WRRGW, pol2-1, pol2-Y831A, and pol32Δ (P<0.0001). The frequency of half-crossovers (HC) was increased in pol3Y-708A, pol3-t, pol31-WRRGW, and pol32Δ (P<0.0001). Note: While this figure shows the fraction of HCs among all DSB repair events, Fig. 2 presents the fraction of colonies that are fully or partially HCs. (TIF) [file pgen.1004119.s001.tif]

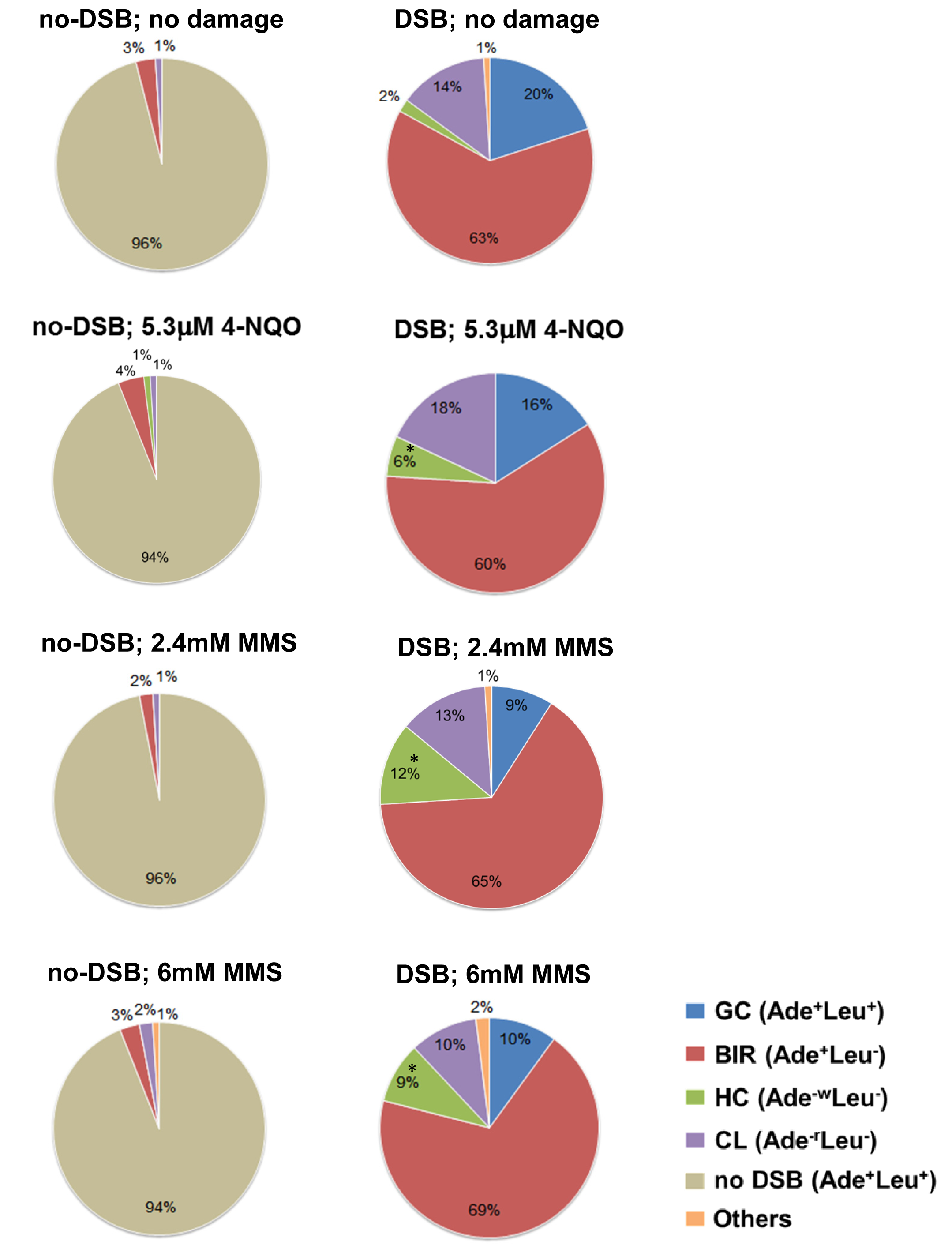

Supplement: Figure S2 — The effect of DNA damaging agents on the distribution of DSB repair outcomes. The fraction (%) of various DSB repair outcomes was determined similarly to [12] following DSB induction in AM1003 and its repair in the presence of MMS or 4-NQO. The data are based on 3 to 4 independent experiments for different conditions. A statistically significant difference from the distribution of DSB repair events in the absence of DNA damage is indicated by asterisk. The frequency of HC was significantly increased following exposure to MMS and 4-NQO (P<0.0001). For no-DSB control, the experiments were performed similarly to [12], but without addition of galactose. Asterisks indicate a statistically significant change as compared to the no-damage control. Note: While this figure shows the fraction of HCs among all DSB repair events, Fig. 2 presents the fraction of colonies that are fully or partially HCs. (TIF) [file pgen.1004119.s002.tif]

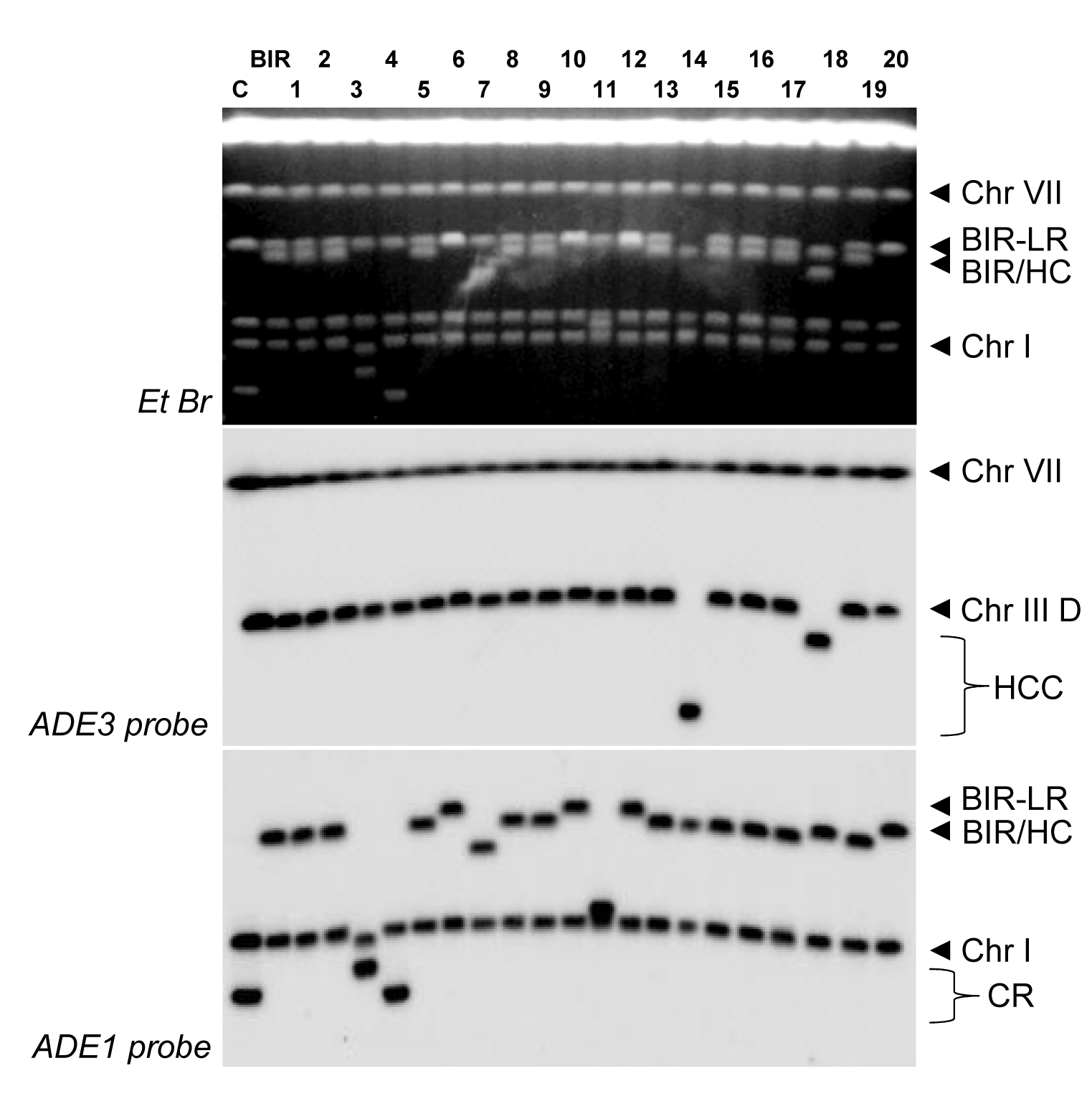

Supplement: Figure S3 — Structural analysis of Ade+Leu− DSB repair outcomes in pol3-t mutants. PFGE analysis of Ade+Leu− DSB repair events obtained from multi-sectored colonies in pol3-t mutants. Ethidium bromide-stained gel (top), Southern blot analysis of PFGE gel using ADE3-specific (middle) and ADE1-specific (bottom) probes are shown. Lane labeled “C”: no-DSB control. Lane labeled “BIR”: DNA from a known BIR outcome. (TIF) [file pgen.1004119.s003.tif]

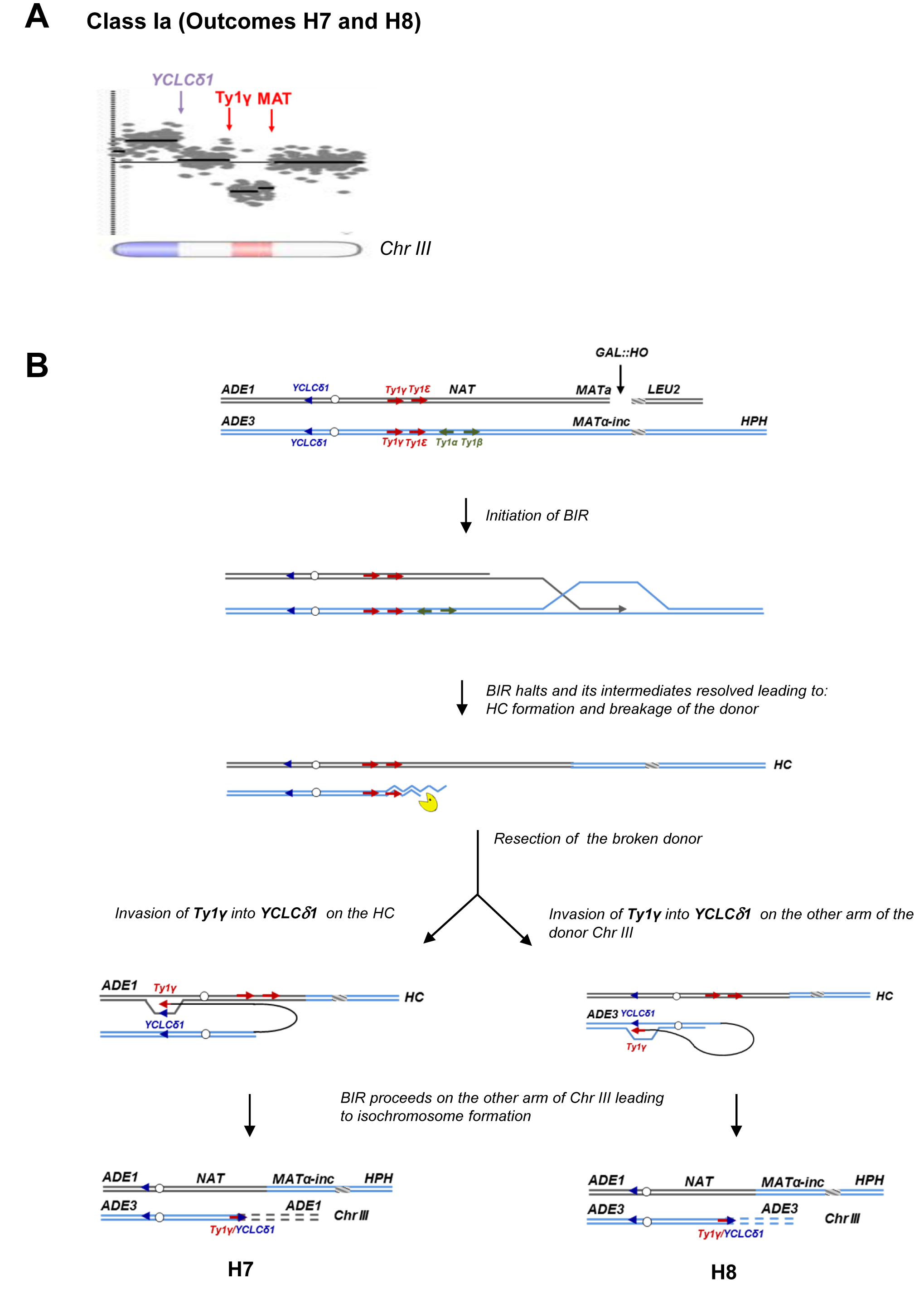

Supplement: Figure S4 — Structural analysis of HCC outcomes H7 and H8. (A) Array-CGH analysis of HCC outcomes H7 and H8 (Class Ia) shows a deletion (red) in Chr III (between FS1 (Ty1γ; 150235 bp position) and MAT and a duplication (blue) of Chr III sequences located centromere-distal to YCLCδ1 (83055 bp position)). (B) Molecular mechanism explaining the formation of H7 and H8. (TIF) [file pgen.1004119.s004.tif]

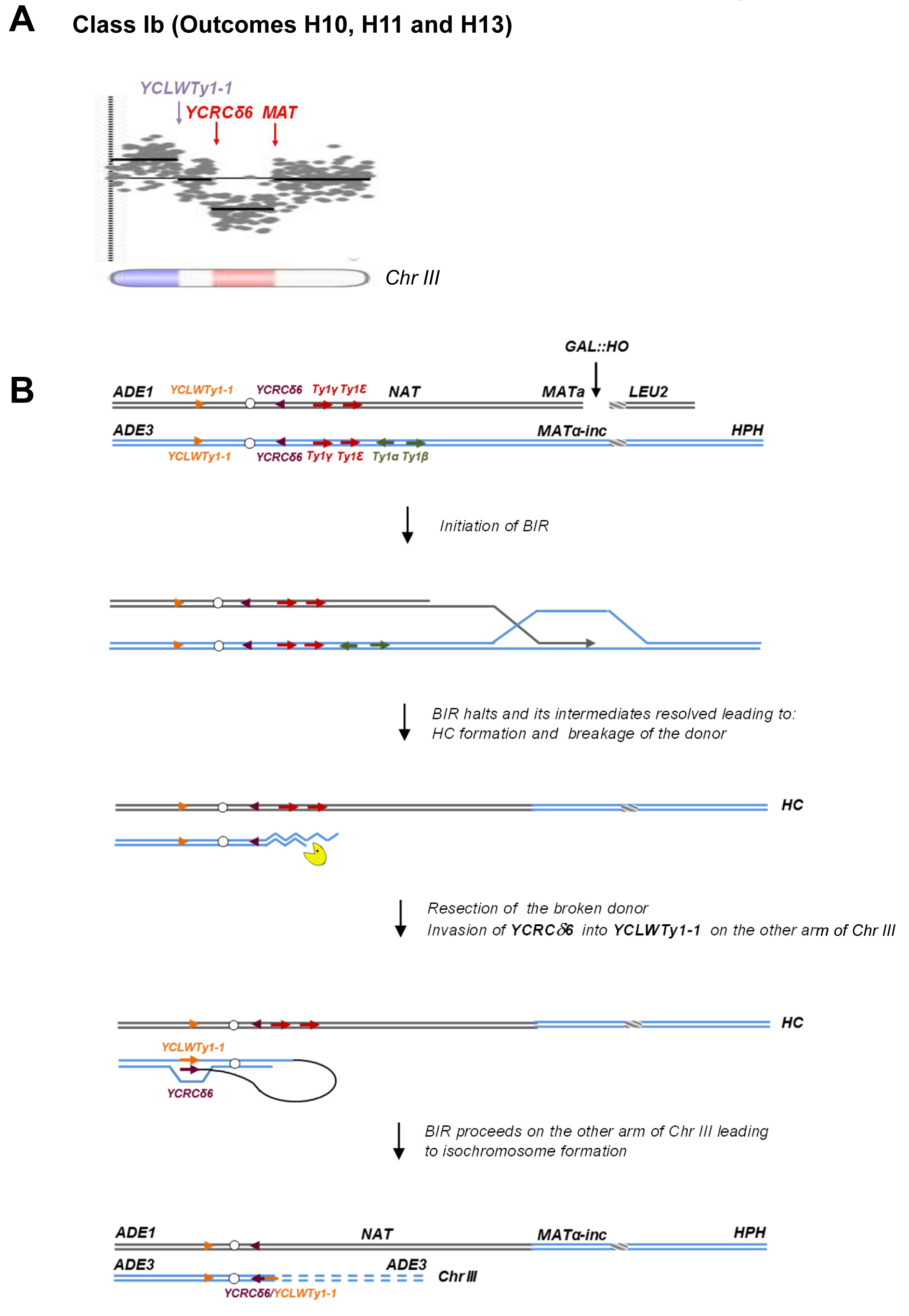

Supplement: Figure S5 — Structural analysis of HCC outcomes H10, H11 and H13. (A) Array-CGH analysis of HCC outcomes H10, H11 and H13 (Class Ib) shows a deletion (red) in Chr III (between YCRCδ6; 124250 bp position) and MAT; and a duplication (blue) of Chr III sequences located centromere-distal to YCLWTy1-1, which corresponds to YCLWδ15 in SGD (83110 bp position). (B) Molecular mechanism explaining the formation of H10, H11, and H13. (TIF) [file pgen.1004119.s005.tif]

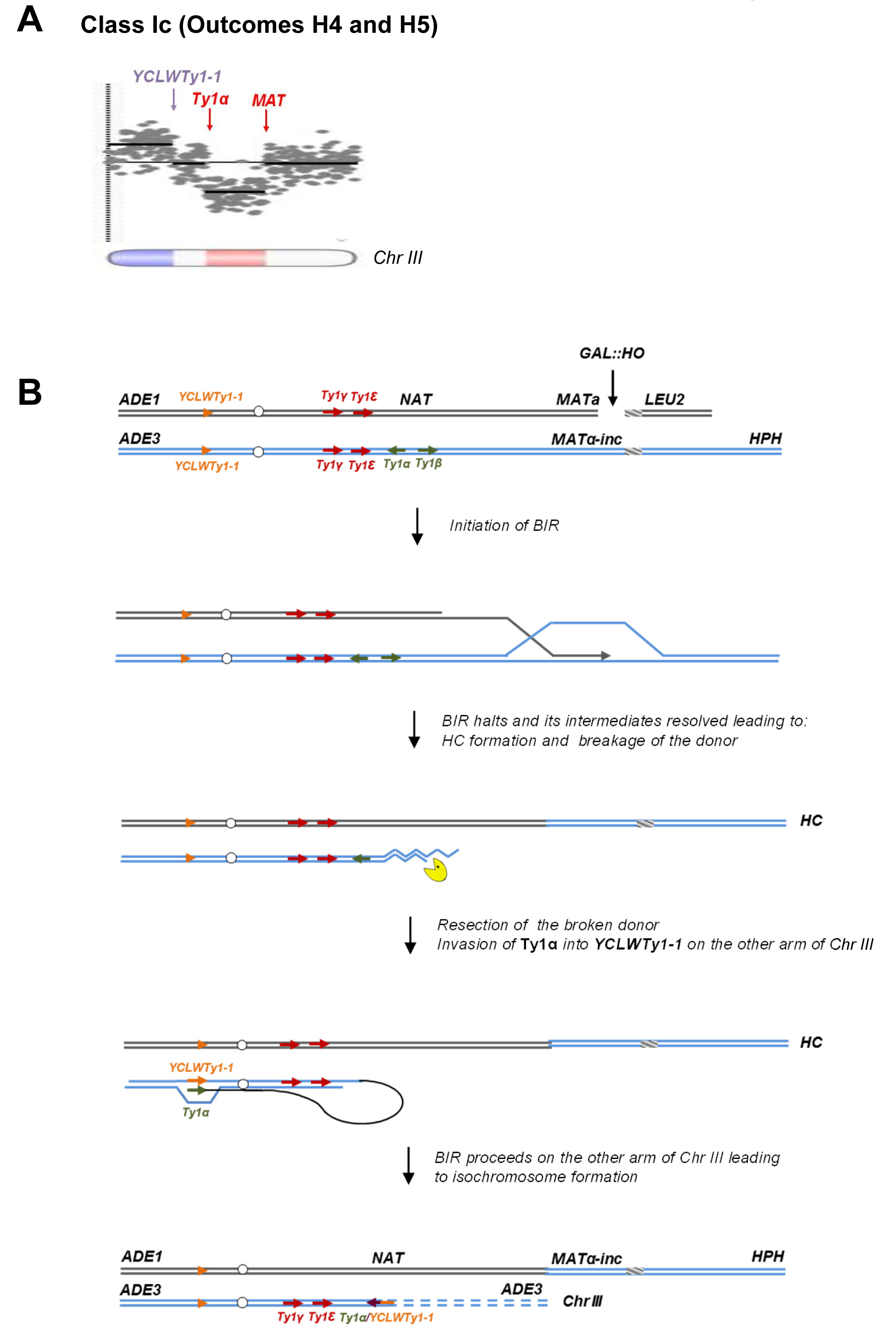

Supplement: Figure S6 — Structural analysis of HCC outcomes H4 and H5. (A) Array-CGH analysis of HCC outcomes H4 and H5 (Class Ic) shows a deletion (red) in Chr III (between FS2; 169419 bp position) and MAT; and a duplication (blue) of Chr III sequences located centromere-distal to YCLWTy1-1, which corresponds to YCLWδ15 in SGD (83110 bp position). (B) Molecular mechanism explaining the formation of H4 and H5. (TIF) [file pgen.1004119.s006.tif]

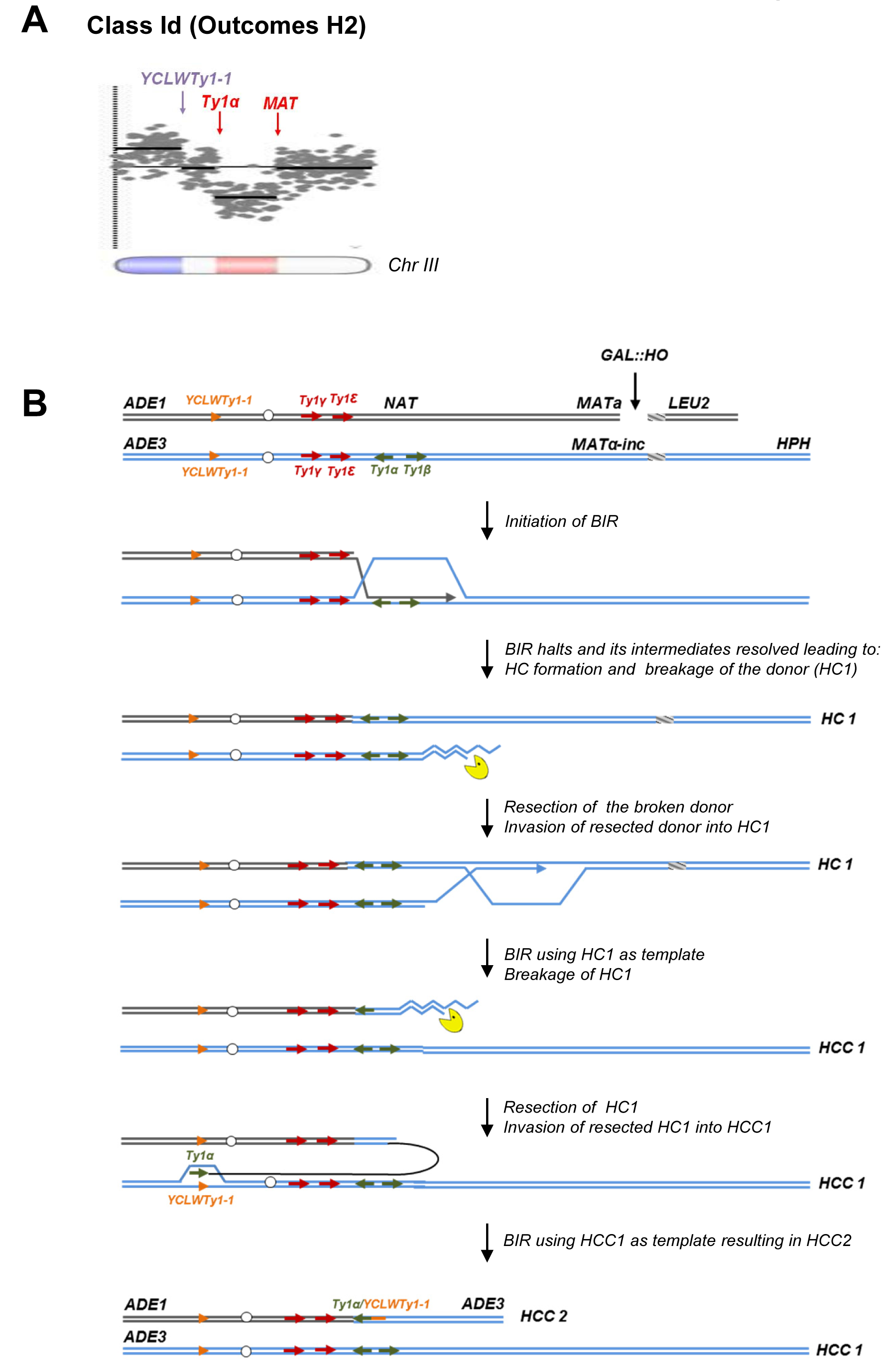

Supplement: Figure S7 — Structural analysis of HCC outcome H2. (A) Array-CGH analysis of HCC outcome H2 (Class Id) shows a deletion (red) in Chr III (between FS2; 169419 bp position) and MAT; and a duplication (blue) of Chr III sequences located centromere-distal to YCLWTy1-1, which corresponds to YCLWδ15 in SGD (83110 bp position). (B) Molecular mechanism explaining the formation of H2. (TIF) [file pgen.1004119.s007.tif]

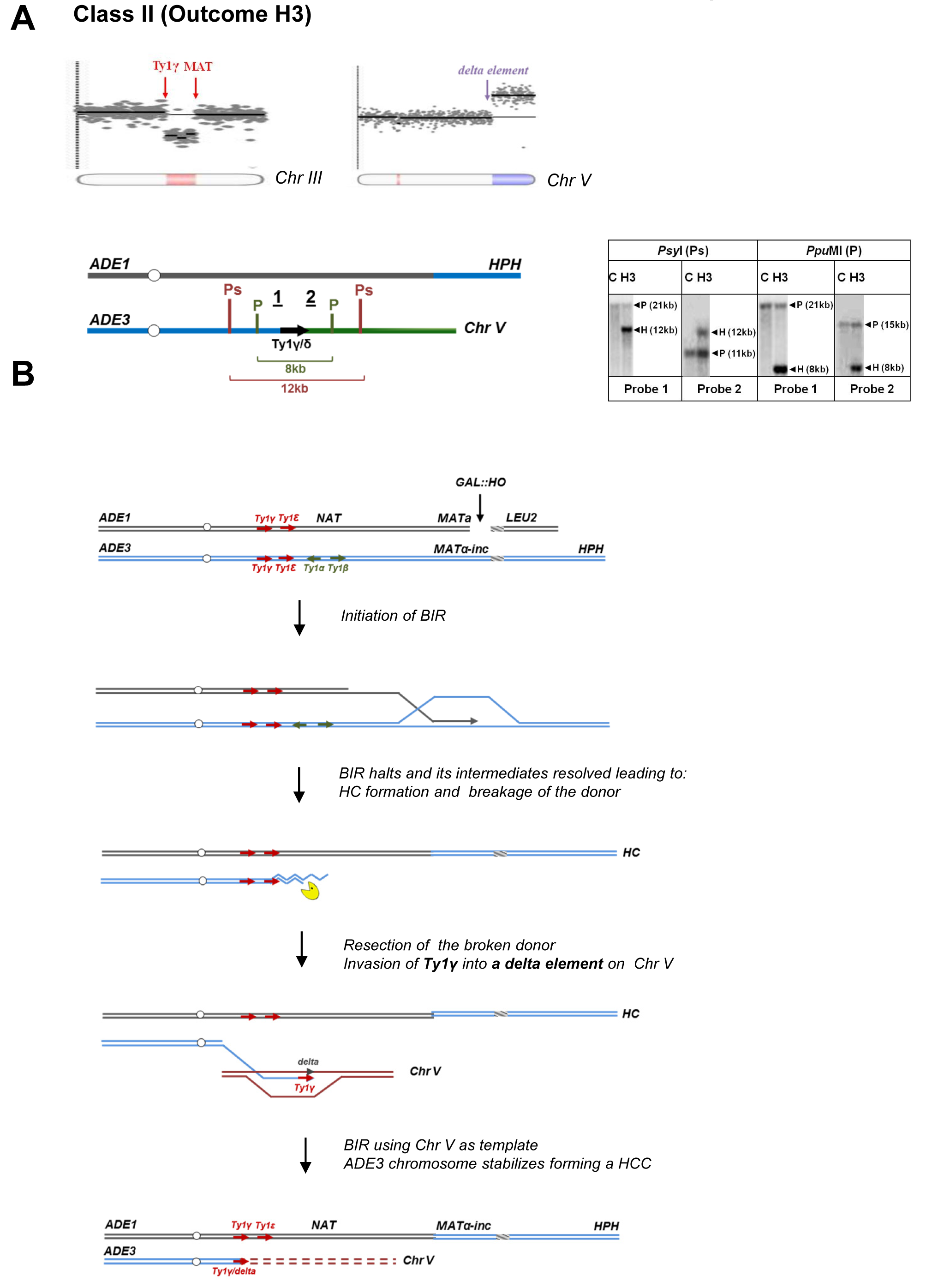

Supplement: Figure S8 — Structural analysis of HCC outcome H3. (A) Array-CGH analysis of HCC outcome H3 (Class II) shows a deletion (red) in Chr III (between FS1 (Ty1γ; 149482 bp position) and MAT; and a duplication (blue) of sequences in Chr V (located centromere-distal to a delta element located close to YERCδ16 (435946 bp position)). Underlined numbers (1 and 2) indicate the positions of probes used for Southern hybridization. The positions of PsyI (Ps) and PpuMI (P) restriction sites are indicated. The Southern blot of H3 genomic DNA digested with PsyI and hybridized to Probes 1 and 2 shows a 12-kb-long DNA fragment at the HCC junction. Also, an 8 kb DNA fragment was obtained when H3 was digested with PpuMI and hybridized with Probe 1 and Probe 2. (B) Model explaining molecular mechanism of H3 formation. (TIF) [file pgen.1004119.s008.tif]

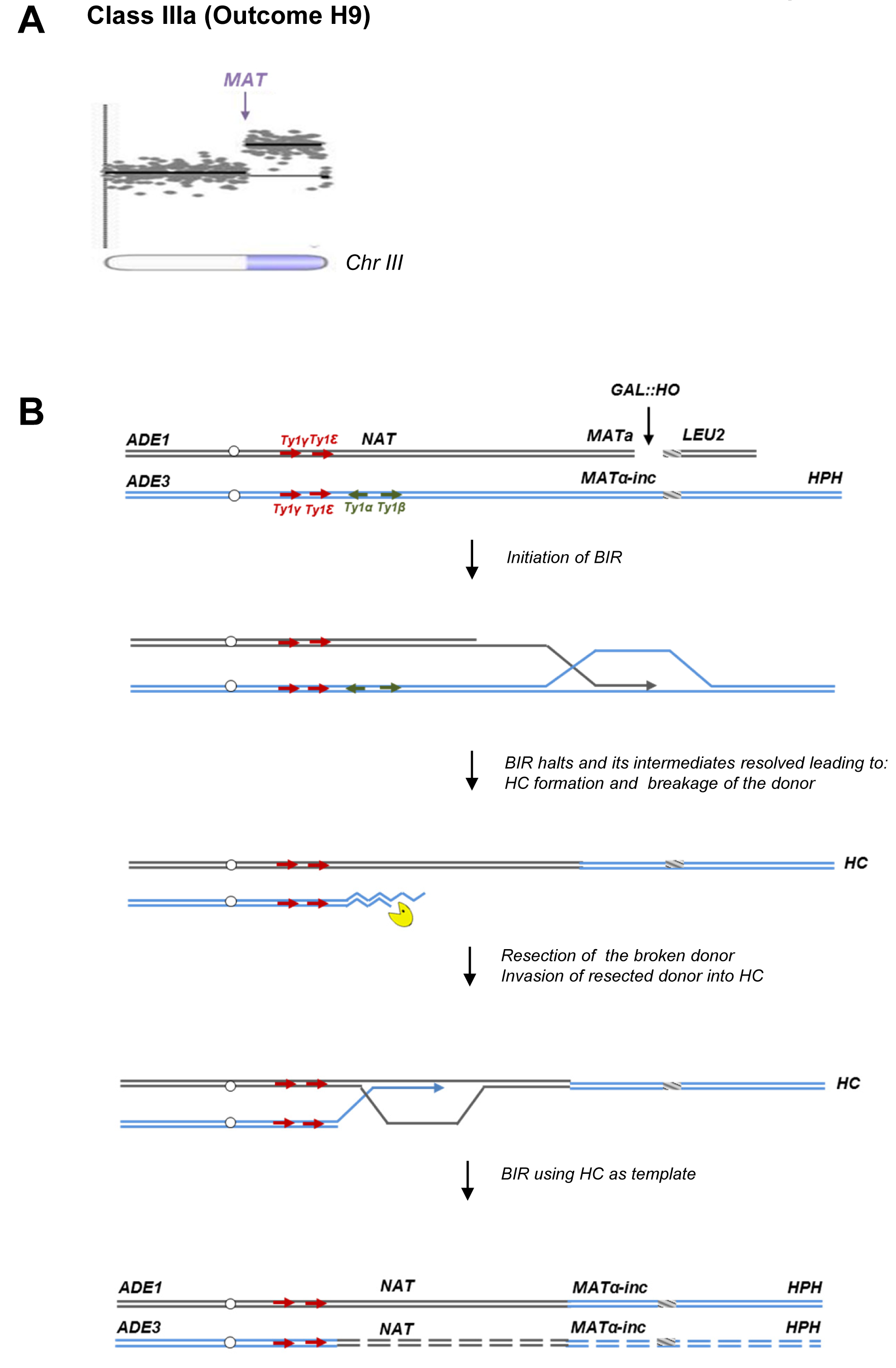

Supplement: Figure S9 — Structural analysis of HCC outcome H9. (A) Array-CGH analysis of HCC outcome H9 (Class IIIa) shows duplication (blue) of Chr III from MAT through the telomeric end. (B) Molecular mechanism explaining the formation of H9. (TIF) [file pgen.1004119.s009.tif]

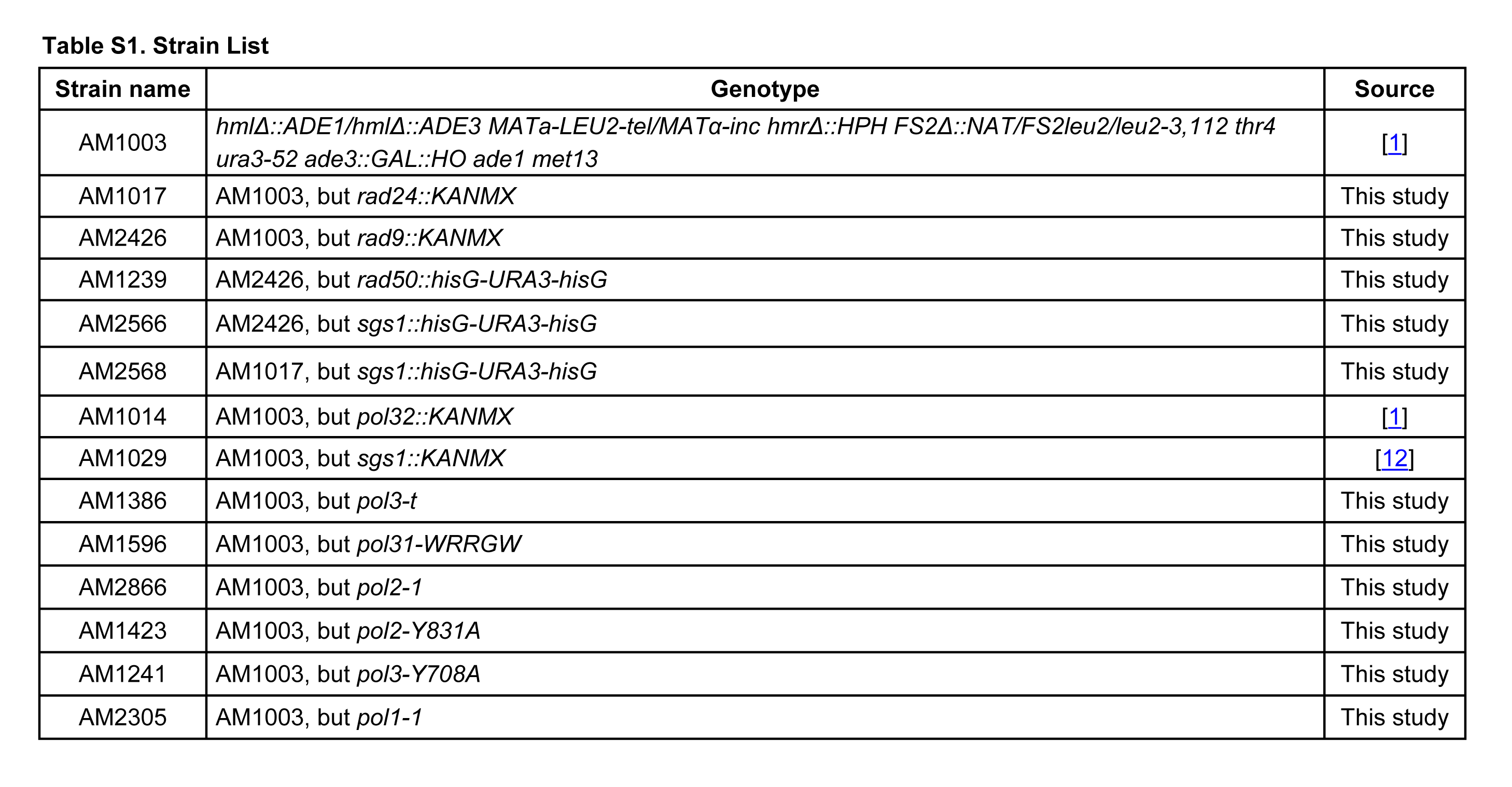

Supplement: Table S1 — Strain list. The list of strains of yeast Saccharomyces cerevisiae that were used in this study. (TIF) [file pgen.1004119.s010.tif]

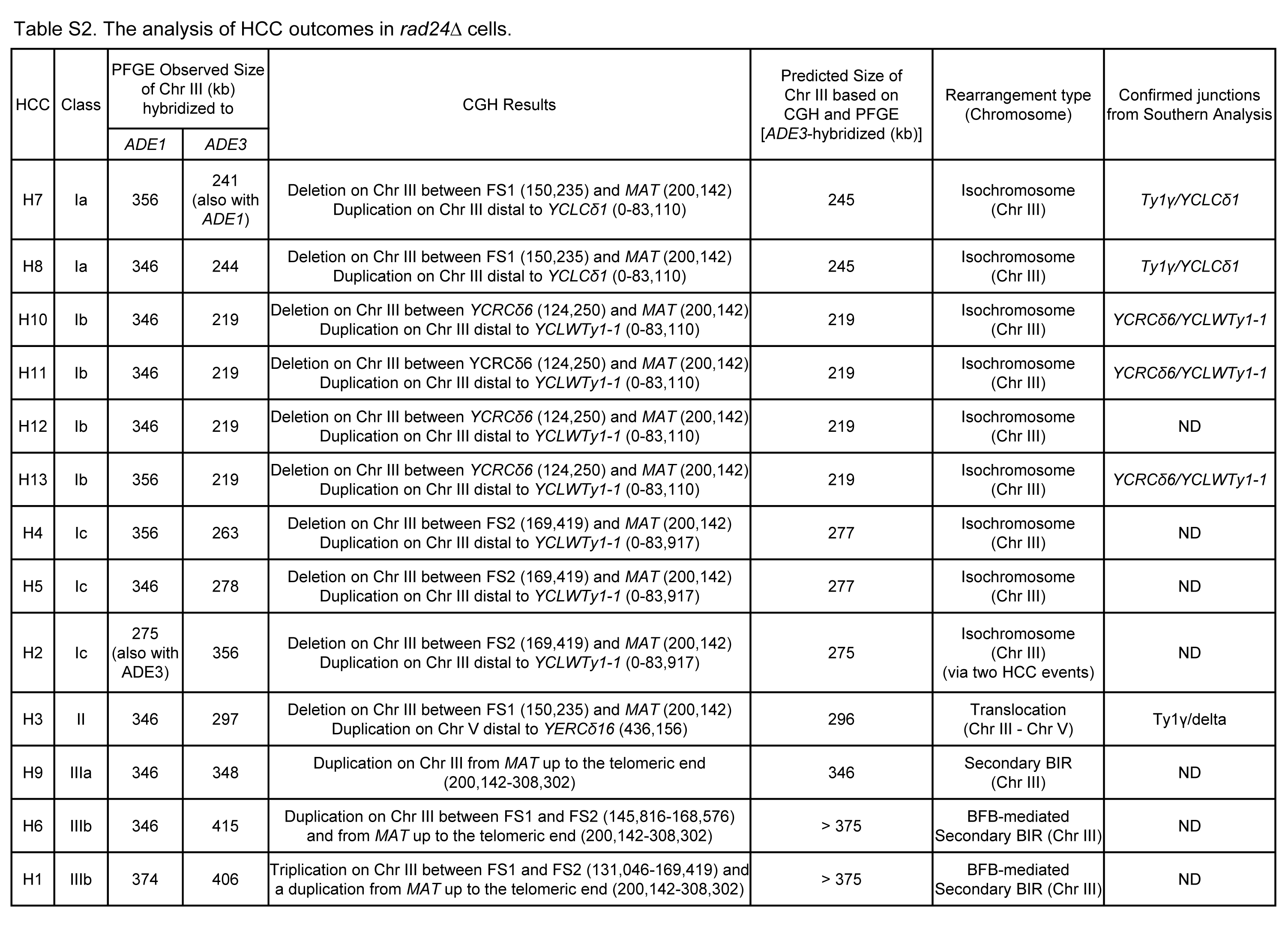

Supplement: Table S2 — The analysis of HCC outcomes in rad24Δ cells. Catalog of half-crossover-initiated cascade DSB repair outcomes that were identified in rad24Δ and characterized by array-CGH and PFGE. BFB: breakage-fusion-bridge cycle. (TIF) [file pgen.1004119.s011.tif]

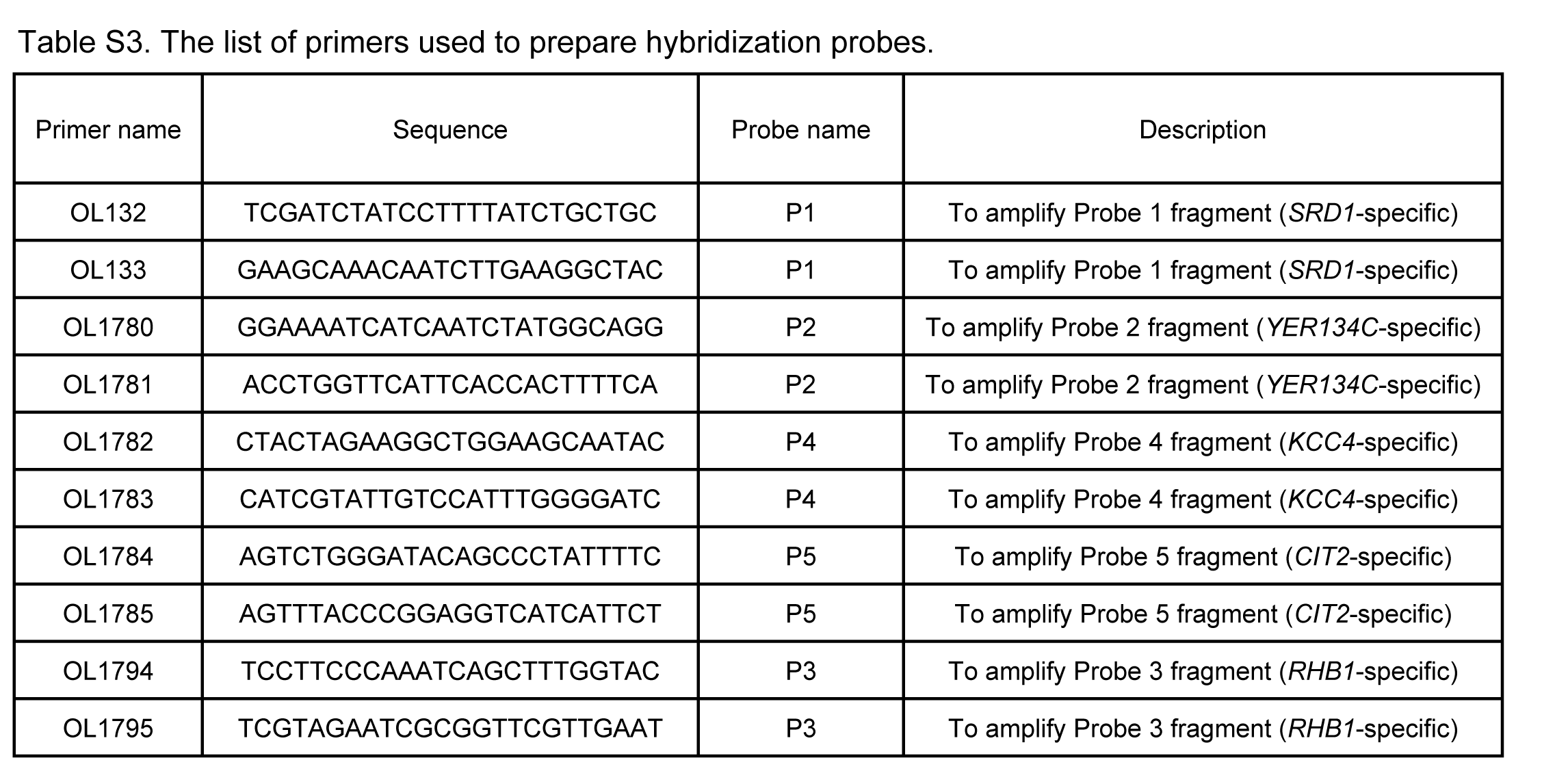

Supplement: Table S3 — The list of primers used to prepare hybridization probes. The 5′-3′ sequences of primers use to prepare hybridization probes by PCR are presented. (TIF) [file pgen.1004119.s012.tif]
