# Supplementary material for: Cascades of Genetic Instability Resulting from Compromised Break-Induced Replication
Source: PLoS Genet. 2014 Feb 27;10(2):e1004119. doi: 10.1371/journal.pgen.1004119 (PMC3937135; doi:10.1371/journal.pgen.1004119)
Supplement: Text S1 — Supplemental Results and Discussion. Description of HCC outcomes that were identified in rad24Δ and characterized by array-CGH and PFGE. The discussion of the mechanisms of their formation. (DOCX) [file pgen.1004119.s013.docx]

Supplemental Results and Discussion.

As described in the main text, we analyzed chromosome rearrangements in HCC outcomes resulting from the repair of *HO*-induced DSBs in the *rad24Δ* strains using both PFGE and array-CGH. Below, we describe additional details of the analysis of these HCCs obtained from AM1017 (*rad24Δ*). The results of this analysis are summarized in Table S2, and Figs. S4-S9.

Characterization of breakpoints in the HCC outcome H3 (Class II)

We propose that the formation of H3 was initiated by invasion of the broken *MAT*a chromosome into the full copy of *MATα*-inc-containing chromosome III, which led to the formation of an HC represented by a 346 kb band that hybridized to the *ADE1*-specific probe (Fig. 7A, Fig. S8). The resulting broken *ADE3*-containing fragment was then resected, leading to the deletion of sequences between *FS1* and *MAT*, and was later stabilized by recombination between Ty1γ(FS1) and a delta element located close to *YERCdelta16* on chromosome V. The predicted size of such a translocation is 296 kb, which is consistent with the size of the band that hybridized to the *ADE3*-specific probe (Figure 7A, Table S2). Our proposed molecular structure was further confirmed through a detailed Southern analysis using the restriction enzymes *PsyI* and *Ppu*MI combined with Probe 1 (FS1-specific) and Probe 2 (specific to the region of chromosome V located centromere-distal to *YERCdelta16*; Fig. S8; see Materials and Methods). Notably, the orientation of the delta elements within Ty1γand *YERCdelta16* is not consistent with the generation of a monocentric translocation; therefore, we propose that recombination occurred between Ty1γand an un-annotated delta element located close to *YERCdelta16* but in the opposite orientation. This recombination between chromosome III and V could proceed by BIR or by a half-crossover event. In the latter case, it would continue the cascade of genomic instability.

Characterization of the HCC outcomes H4 and H5 (Class Ic)

We propose that the formation of H4 and H5 was initiated by invasion of the broken *MAT*a chromosome into the full copy of *MATα*-inc-containing chromosome III, which led to the formation of an HC. The only difference was that in the case of H5, the invasion occurred centromere-distal to FS2, thus leading to the formation of HC represented by a 346 kb band while in the case of H4, the invasion occurred centromere-proximal to FS2 which resulted in the formation of a 356 kb HC band (Fig. 7A; Fig. S6). The broken *ADE3*-containing fragment resulted from HC formation was then resected, leading to the deletion of sequences between *FS2* and *MATα*-inc, and was later stabilized by recombination between Ty1α (FS2) and a delta element of *YCLWTy1-1*, which corresponds to *YCLWδ15* in SGD (83110 bp position) in the left arm of chromosome III. The predicted size of such a translocation is 277 kb, which is consistent with the size of the band that hybridized to the *ADE3*-specific probe in H5 (Figure 7A, Table S2). The reduced size of *ADE3-*hybridized bands observed in H4 (263 kb) might result from recombination between delta elements of Ty1α of FS2 and *YCLWTy1-1* in such a way that it resulted in deletion of Ty1 at the junction.

Characterization of the HCC outcomes H2 (Class Id)

We propose that the formation of H2 was initiated by invasion of the broken *MAT*a chromosome into the full copy of *MATα*-inc-containing chromosome III in the region between FS1 and FS2, which led to the formation of HC1 (Fig. S7). Next, the broken *ADE3*-containing fragment was resected and invaded into the HC1 in the chromosomal region located centromere distal to FS2, which led to the formation of another HC molecule (HCC1). The resulting broken *ADE1*-containing fragment was stabilized by strand invasion of Ty1α (FS2) into *YCLWTy1-1* of the HCC1 followed by BIR that proceeded to the end of the chromosome.

Characterization of the HCC outcome H6 (Class IIIb)

Array-CGH analysis of the HCC outcome H6 (Class IIIb) demonstrated a duplication of chromosome III sequences from *MAT* to the telomere, which was similar to all normal BIR. In addition, duplication between FS1 and FS2 was observed. However, the PFGE analysis detected a change in the donor chromosome, which became longer than the BIR product (415 kb) (Fig. 7A, Table S2). Due to the change in the donor chromosome, it was placed in the HCC category, and we propose that it was also formed by a cascade of events. Similar to classes I and II, its formation was also triggered by a breakage in the donor chromosome resulting from the formation of a HC. Further, we propose that the broken donor chromosome was stabilized through breakage-fusion bridge (BFB) cycle (similar to [[1-3](#_ENREF_1)]), where simultaneous breakage of two donor sister chromatids resulted in the formation in the inverted dicentric dimer, which initiated a BFB cycle, followed by stabilization of a broken fragment by BIR using HC as a template.

Characterization of the HCC outcome H1 (Class IIIb)

Array-CGH analysis of the HCC outcome H1 (Class IIIb) demonstrated a duplication of chromosome III sequences from *MAT* to the telomere, which was similar to all normal BIR. In addition, a triplication between FS1 and FS2 was observed. The PFGE analysis detected a change in the donor chromosome, which became longer than the BIR product (407 kb) (Fig. 7A, Table S2), and the change of the size of the recipient chromosome, which became 374 kb long. We propose that H1 represents a complex event, and several steps contributed to its formation. Specifically, similar to H6, the formation of H1 was triggered by a breakage in the donor chromosome resulting from the formation of a HC. Also, the donor likely stabilized via a BFB cycle. In addition, it is possible that a BFB cycle or a template switching event contributed to the formation of the original HC, which resulted in its increased size (374kb).

Supplemental References

1. Lemoine FJ, Degtyareva NP, Lobachev K, Petes TD (2005) Chromosomal translocations in yeast induced by low levels of DNA polymerase a model for chromosome fragile sites. Cell 120: 587-598.

2. Vanhulle K, Lemoine FJ, Narayanan V, Downing B, Hull K, et al. (2007) Inverted DNA repeats channel repair of distant double-strand breaks into chromatid fusions and chromosomal rearrangements. Mol Cell Biol 27: 2601-2614.

3. Narayanan V, Mieczkowski PA, Kim HM, Petes TD, Lobachev KS (2006) The pattern of gene amplification is determined by the chromosomal location of hairpin-capped breaks. Cell 125: 1283-1296.
